# SARS-CoV-2 spike-FLIPr fusion protein plus lipidated FLIPr protects against various SARS-CoV-2 variants in hamsters

**DOI:** 10.1128/jvi.01546-23

**Published:** 2024-02-01

**Authors:** Ming-Shu Hsieh, Chia-Wei Hsu, Hung-Chun Liao, Chang-Ling Lin, Chen-Yi Chiang, Mei-Yu Chen, Shih-Jen Liu, Ching-Len Liao, Hsin-Wei Chen

**Affiliations:** 1 National Institute of Infectious Diseases and Vaccinology, National Health Research Institutes, Miaoli, Taiwan; 2 Graduate Institute of Biomedical Sciences, China Medical University, Taichung, Taiwan; 3 Graduate Institute of Medicine, Kaohsiung Medical University, Kaohsiung, Taiwan; University of North Carolina at Chapel Hill, Chapel Hill, North Carolina, USA

**Keywords:** FLIPr, mucosal immunity, neutralizing antibodies, SARS-CoV-2, T-cell response, variants, vaccine development

## Abstract

Vaccine-induced mucosal immunity and broad protective capacity against various severe acute respiratory syndrome coronavirus 2 (SARS-CoV-2) variants remain inadequate. Formyl peptide receptor-like 1 inhibitory protein (FLIPr), produced by *Staphylococcus aureus*, can bind to various Fcγ receptor subclasses. Recombinant lipidated FLIPr (rLF) was previously found to be an effective adjuvant. In this study, we developed a vaccine candidate, the recombinant Delta SARS-CoV-2 spike (rDS)-FLIPr fusion protein (rDS-F), which employs the property of FLIPr binding to various Fcγ receptors. Our study shows that rDS-F plus rLF promotes rDS capture by dendritic cells. Intranasal vaccination of mice with rDS-F plus rLF increases persistent systemic and mucosal antibody responses and CD4/CD8 T-cell responses. Importantly, antibodies induced by rDS-F plus rLF vaccination neutralize Delta, Wuhan, Alpha, Beta, and Omicron strains. Additionally, rDS-F plus rLF provides protective effects against various SARS-CoV-2 variants in hamsters by reducing inflammation and viral loads in the lung. Therefore, rDS-F plus rLF is a potential vaccine candidate to induce broad protective responses against various SARS-CoV-2 variants.

**IMPORTANCE:**

Mucosal immunity is vital for combating pathogens, especially in the context of respiratory diseases like COVID-19. Despite this, most approved vaccines are administered via injection, providing systemic but limited mucosal protection. Developing vaccines that stimulate both mucosal and systemic immunity to address future coronavirus mutations is a growing trend. However, eliciting strong mucosal immune responses without adjuvants remains a challenge. In our study, we have demonstrated that using a recombinant severe acute respiratory syndrome coronavirus 2 (SARS-CoV-2) spike-formyl peptide receptor-like 1 inhibitory protein (FLIPr) fusion protein as an antigen, in combination with recombinant lipidated FLIPr as an effective adjuvant, induced simultaneous systemic and mucosal immune responses through intranasal immunization in mice and hamster models. This approach offered protection against various SARS-CoV-2 strains, making it a promising vaccine candidate for broad protection. This finding is pivotal for future broad-spectrum vaccine development.

## INTRODUCTION

The spread of the severe acute respiratory syndrome coronavirus 2 (SARS-CoV-2) virus in late 2019 triggered a pandemic of severe acute respiratory syndrome, which continues to impact the world today (1–3). As an RNA virus with a large genome, SARS-CoV-2 has a relatively high rate of mutations, with various variants emerging after over 2 years of continuous spread. Mutations in the spike (S) protein have altered the virulence, infectivity, and antigenicity of the virus (4–7). Even though several COVID-19 vaccines have received approval for emergency use during the pandemic (8), there is still a need for more broadly protective vaccines to help combat breakthrough infections caused by a decline in immunity to newly emerging SARS-CoV-2 variants.

To effectively manage and protect against pathogen invasions through mucosal surfaces, including SARS-CoV-2, it is believed that activation of both the systemic and mucosal branches of the immune system is necessary. The combination of IgG and IgA antibodies is considered crucial for blocking the entry of pathogens into the host through mucosal surfaces (9–11). The role of neutralizing antibodies in protecting against SARS-CoV-2 infection is well established, and they have been proposed as a useful benchmark for evaluating vaccine efficacy in clinical trials and for surveying immunity levels in populations (12–14). In addition, non-neutralizing antibodies (15–17) and T cells (18–20) are believed to play a role in alleviating severe illness and resolving infections through complementary immune mechanisms. Therefore, in the ongoing battle against the SARS-CoV-2 epidemic, it is essential to implement immunization strategies that can produce systemic antibody responses, humoral immunity at the mucosal entry points of the virus, and robust T-cell responses.

We previously developed a vaccine platform that utilizes formyl peptide receptor-like 1 inhibitory protein (FLIPr) as a vector to enhance the immune response by targeting antigens to Fcγ receptors (FcγRs) dendritic cells (DCs) (21). Furthermore, we demonstrated that intranasal administration of antigen-FLIPr fusion protein alone efficiently delivers antigens to DCs in nasal lymphoid tissue and then elicits antigen-specific CD4^+^ and CD8^+^ T-cell responses as well as IgG and IgA antibodies in the circulatory system and IgA antibodies in mucosal tissue (22). These findings indicate that using this vaccine platform offers benefits for the development of SARS-CoV-2 vaccines.

Newly developed SARS-CoV-2 vaccines can take consensus sites of majority variants (23) or use conserved S2 subunit of the spike protein (24). Alternatively, the potency of existing vaccines with the original strain antigens can be increased by adjuvant, resulting in a robust immune response that can effectively neutralize even variants that evade the immune system (23, 25–28). Numerous studies have indicated that toll-like receptor (TLR) agonists are effective as mucosal adjuvants (29–31). In particular, intranasal immunization of mice with antigen-containing synthetic lipopeptide (a TLR-2 agonist) not only enhances systemic but also mucosal immune responses (32, 33). In the past, we developed a novel technology to express recombinant lipoprotein in high yields using an *Escherichia coli*-based system (34). These recombinant lipoproteins can be classified as TLR-2 agonists (35, 36). Recently, we produced recombinant lipidated FLIPr (rLF) and demonstrated that rLF is an effective adjuvant (37). Formulating an antigen with rLF can lead to long-lasting antigen-specific immune responses and can enhance both mucosal and systemic antibody responses, as well as broad-spectrum T-cell responses in mice. These findings suggest that rLF is a favorable adjuvant for SARS-CoV-2 vaccines.

In the present study, we prepared a recombinant Delta SARS-CoV-2 spike (rDS)-FLIPr fusion protein (rDS-FLIPr) and evaluated the immunogenicity of rDS-FLIPr alone or with rLF. We demonstrated that intranasal administration of rDS-FLIPr plus rLF further promoted systemic and mucosal immune responses. Importantly, rDS-FLIPr plus rLF could induce neutralizing antibodies against different variants of SARS-CoV-2 and provide superior protection in the hamster challenge model. These results provide important information for future clinical studies of rDS-FLIPr plus rLF as a vaccine formulation against SARS-CoV-2.

## RESULTS

### Production and characterization of rDS and rDS-F

**Fig 1:**
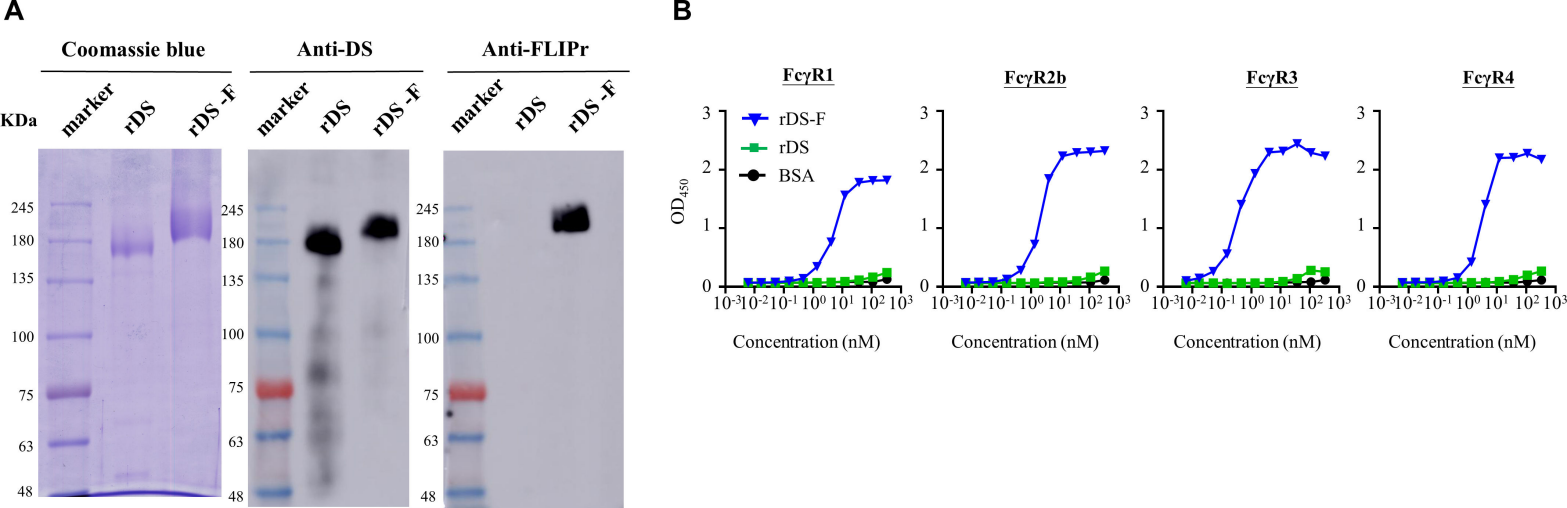
Production and characterization of rDS and rDS-F. Purified rDS and rDS-F were examined by 10% reducing SDS-PAGE followed by (**A**) Coomassie blue staining and immunoblotting with anti-DS or anti-FLIPr antibodies. (**B**) rDS-F but not rDS can bind to various Fcγ receptors. Fcγ receptor-1, -2b, -3, or -4 was coated on 96-well plates (0.5 µg/well). After blocking, a graded concentration of biotin-conjugated rDS or rDS-F was added to each well followed by incubation at room temperature for 2 hours. The bovine serum albumin served as a negative control. After washing unbound protein, HRP-conjugated streptavidin was added for the detection of binding protein. A substrate, 3,3',5,5'-tetramethylbenzidine, was added for color development. The absorbance was measured with an ELISA reader at 450 nm. The results are shown from one representative experiment, with three replicates for each group.

The rDS and rDS-F were produced using CHO cell-expressed systems. After purification, both proteins were subjected to 10% SDS-PAGE and stained with Coomassie blue or examined by immunoblotting using anti-spike protein or anti-FLIPr antibodies (Fig. 1A; raw images in Fig. S1). While both rDS and rDS-F were detected by anti-spike antibodies, only rDS-F was recognized by anti-FLIPr antibodies. These results confirmed that the purified proteins correspond to rDS and rDS-F, respectively.

To investigate the interaction between rDS-F and FcγRs, we performed a capture ELISA. The results showed that rDS-F was captured by FcγR1, FcγR2b, FcγR3, and FcγR4 in a dose-dependent manner (Fig. 1B). On the other hand, there was little to no interaction between rDS and FcγRs, even at concentrations >100 nM. These findings provide further evidence that rDS-F is capable of binding to different mouse FcγR isoforms.

### Intranasal vaccination of rDS-F plus rLF increases antigen transmucosal uptake by antigen-presenting cells and promotes DCs’ activation *in vivo*

We previously demonstrated that antigens fused with FLIPr (22) or combined with rLF (37) can improve antigen uptake by antigen-presenting cells (APCs) in nasal-associated lymphoid tissue (NALT). Based on these findings, we hypothesized that adding rLF to rDS-F could further enhance its uptake across the nasal mucosa and increase its capture by DCs. To investigate this hypothesis, groups of C57BL/6JNarl mice were intranasally administered 20 µg of rDS with or without 10 µg rLF and 20 µg of rDS-F with or without 10 µg rLF. These experiments included phosphate-buffered saline (PBS)-administered mice as controls.

**Fig 2:**
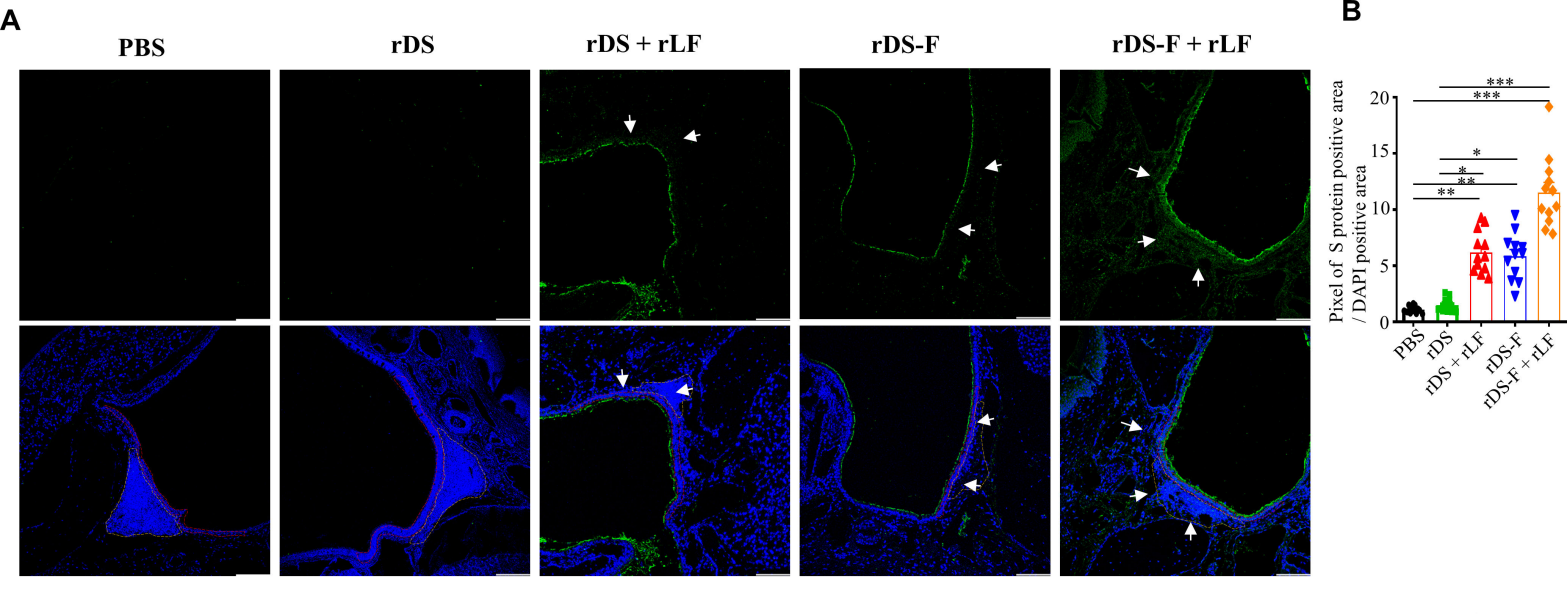
Intranasal administration of rDS-F plus rLF increases the deposition of rDS-F on the nasal epithelium and enhances its entry into NATL. PBS, rDS, rDS plus rLF, rDS-F, or rDS-F plus rLF were intranasally administered, and the nasal cavity was collected after 1 hour. rDS and rDS-F staining (green) in NALT was performed using an anti-SARS-CoV-2 spike protein antibody following the Alexa Fluor488-conjugated secondary antibody and the representative image was presented in panel A (top panel). Cell nucleus was stained with DAPI (blue) to indicate the epithelial cells of nasal and NALT cells. Representative DAPI-staining images were merged with the S protein-staining images (A, bottom panel). The red dotted lines and yellow dotted lines indicate the nasal epithelial layer and NALT, respectively. The white arrows indicate the penetration of rDS or rDS-F into tissue. Images were obtained from single optical sections acquired by means of confocal microscopy (scale bar = 100 µm). The amount of S protein deposited in a single field was calculated by the total pixel of S protein positive area (green fluorescence) and normalized to the total cell area (blue fluorescence). Cumulative data from four mice per group are summarized in panel **B**. The data are presented as the means ± SEM (*n* = 12 fields/group). Statistical significance was determined using the Kruskal-Wallis test with Dunn’s multiple comparison test. *P < 0.05, **P < 0.01, and ***P < 0.001.

The nasal mucosal tissues were collected 1 hour after administration, and immunofluorescent staining was used to evaluate the presence of the antigen. Representative images are shown in Fig. 2A. Mice administered with PBS or rDS showed background levels. In comparison to mice that received rDS administration alone, the administration of rDS plus rLF increased the deposition of the antigen in the nasal mucosa. It was also observed that the administration of rDS-F not only increased the deposition of the antigen in the nasal mucosa but also facilitated its penetration into the tissues. Both effects were further amplified when the mice were administered with rDS-F plus rLF. Antigen-positive areas (green fluorescence) were normalized with the total cell nucleus area (DAPI staining) to represent antigen uptake. Results were summarized from four mice (three fields per mouse) per group (Fig. 2B). These results suggest that the fusion of DS with FLIPr increases rDS-F deposition in the nasal mucosa. Furthermore, the presence of rLF can further enhance these effects.

**Fig 3:**
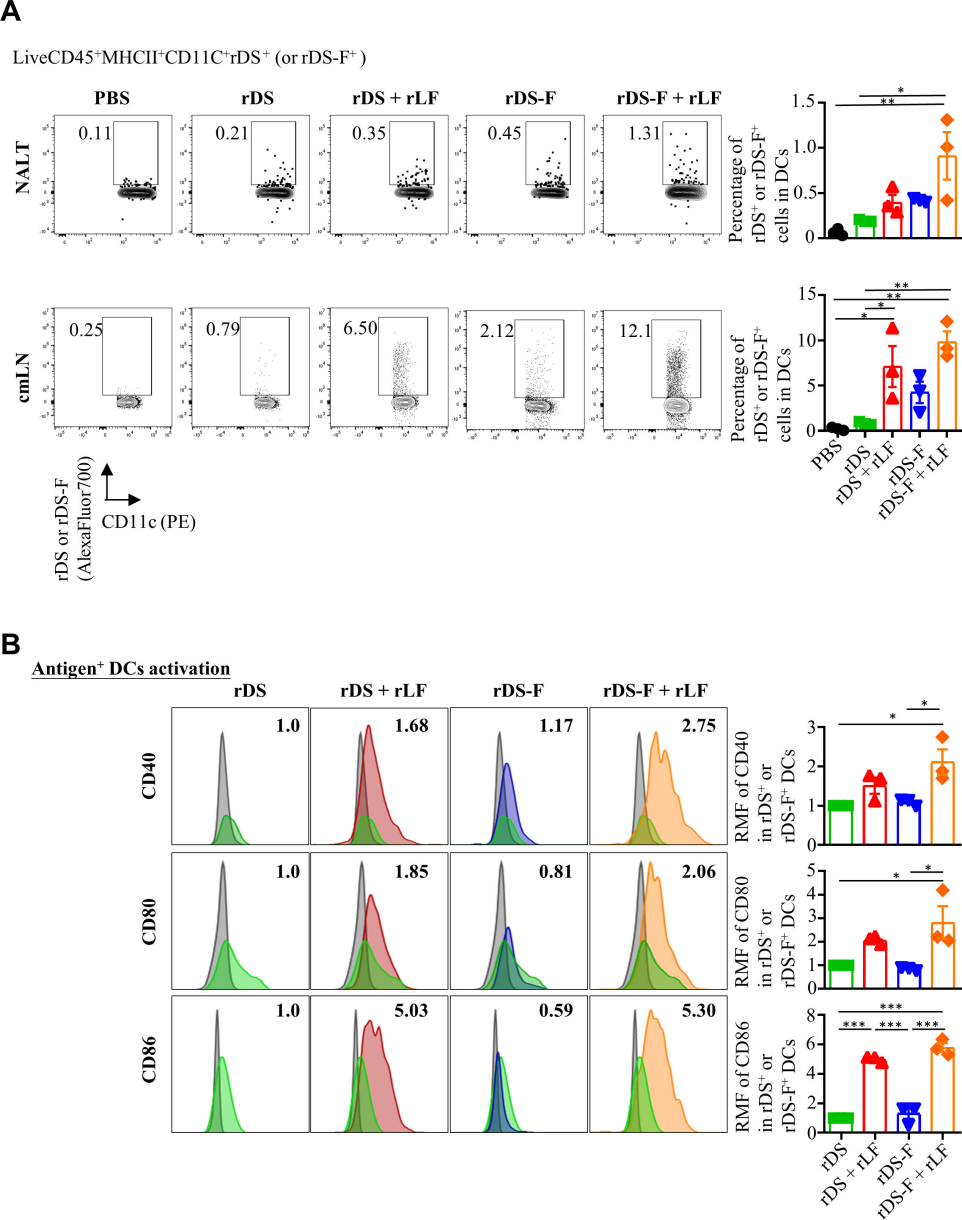
Intranasal vaccination of rDS-F plus rLF effectively enhances antigen delivery to DC and concomitantly promotes DC activation. Groups of C57BL/6JNarl mice were intranasally immunized with 20 µg of Alexa Fluor 700-labeled rDS with or without 10 µg of rLF, or Alexa Fluor 700-labeled rDS-F with or without 10 µg of rLF. Mice administered PBS alone were used as controls. NALT and cmLN were isolated 18 hours after administration. The single-cell suspensions (pooled from three mice) were prepared for flow cytometry analysis of antigen uptake. (**A**) Representative flow cytometry plots display signal gating for rDS or rDS-F and show the percentage of dendritic cells harboring the antigen in NALT (upper-left panel) and cmLN (lower-left panel). Cumulative data from three individual experiments are summarized for NALT (upper-right panel) and cmLN (lower-right panel). (**B**) Representative flow cytometry gating and enumeration expression levels of CD40, CD80, and CD86 on antigen-harboring dendritic cells were analyzed by flow cytometry. The DCs stained with the isotype control IgG were depicted in gray, serving to represent the background fluorescence intensity levels. The mean fluorescence intensity for cells stimulated with rDS alone was defined as the basal expression level. The relative mean fluorescence intensities (the fold change compared to the rDS) were plotted. A representative experiment is shown in the left panel. Cumulative data from three individual experiments are summarized in the right panel. The data are presented as the means ± SEM (*n* = 3). Statistical significance was determined using the ordinary one-way ANOVA followed by Tukey’s multiple comparisons test. *P < 0.05, **P < 0.01, and ***P < 0.001.

The increased antigen uptake in the NALT with FLIPr-fused antigens or the addition of rLF led us to investigate the capture of antigens by DCs. The frequencies of fluorescence-containing CD11c^+^ MHC II^+^ cells in NALT (pooled from three mice) and cervical and mediastinal lymph nodes (cmLNs) were analyzed by flow cytometry 18 hours after injection. The gating strategy is shown in Fig. S2A. In comparison to mice that received rDS administration alone, the administration of rDS plus rLF and rDS-F increased the frequency of fluorescence-containing CD11c^+^ MHC II^+^ cells. Furthermore, the administration of rDS-F plus rLF was the most effective in increasing antigen capture by DCs (Fig. 3A). These results are consistent with the levels of antigen entry into the NALT.

We further investigated the level of activation of antigen-carrying DCs in cmLNs. Compared to DCs in mice administered with rDS alone, DCs in mice administered with rDS plus rLF exhibited relatively higher expression of CD40, CD80, and CD86. On the other hand, rDS-F only slightly increased the expression of CD40. DCs in mice administered with rDS-F plus rLF were the most effective in enhancing the expression of CD40, CD80, and CD86 (Fig. 3B). These results suggest that administration of rLF can promote CD40, CD80, and CD86 expression on DCs. In conclusion, mice that received rDS-F plus rLF not only enhanced antigen uptake by DCs but also increased the migration of antigen-carrying DCs into cmLN with concomitant activation.

### rDS-F adjuvanted with rLF elicits superior germinal center and T follicular helper cell responses after intranasal immunization

**Fig 4:**
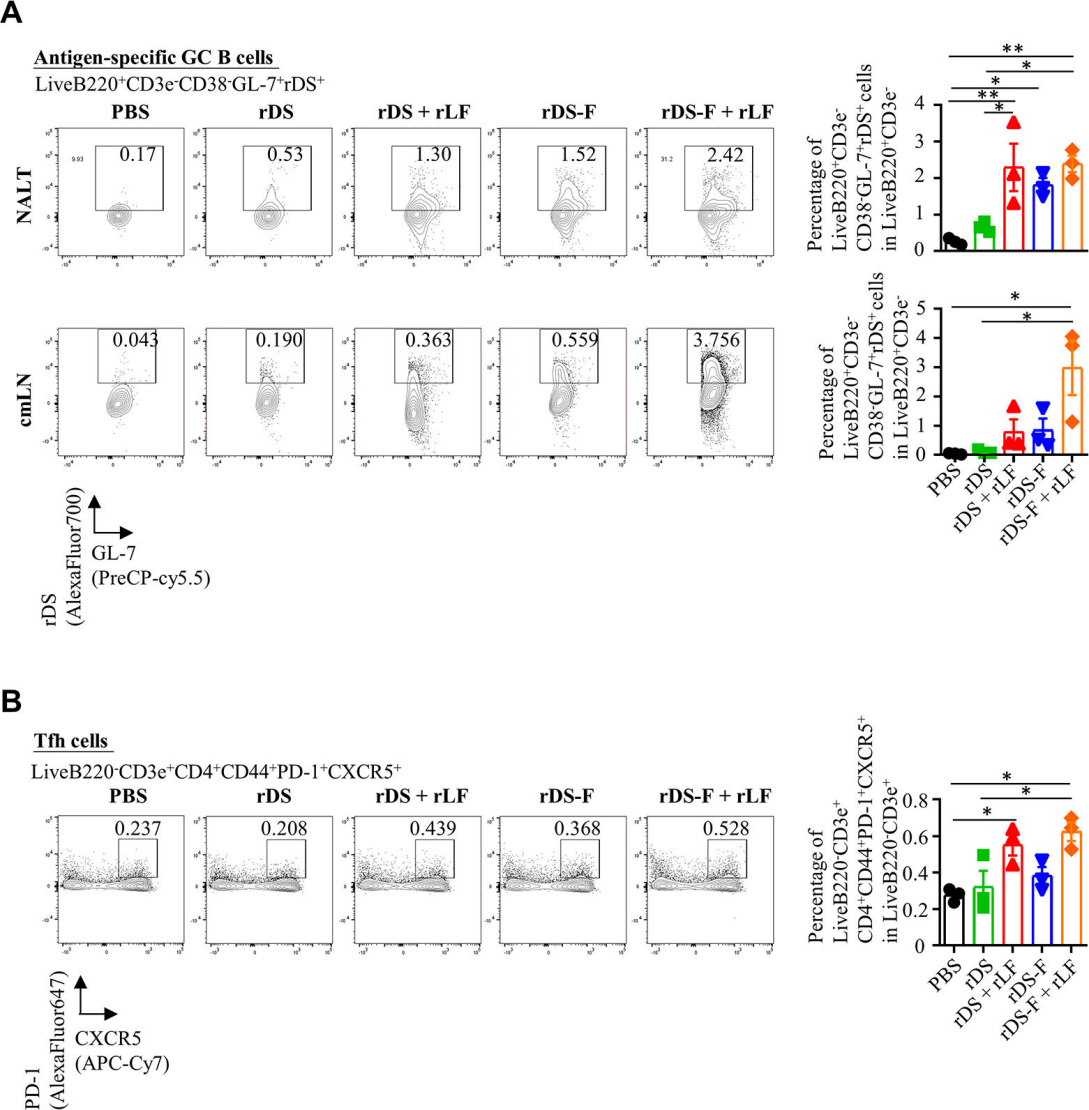
Intranasal vaccination of rDS-F plus rLF enhanced antigen-specific GC B and Tfh cells’ responses in the respiratory-associated lymphoid tissue. Groups of C57BL/6JNarl were immunized with 20 µg of rDS, rDS plus 10 µg rLF, 20 µg rDS-F, or rDS-F plus 10 µg rLF. GC B and Tfh cells’ responses were analyzed by flow cytometry on day 12 after immunization. Representative flow cytometry gating and frequencies of antigen-specific GC B cells (defined as live/B220^+^CD3e^−^/CD38^−^GL-7^+^/rDS^+^ cells) in NALT and cmLN are shown (A, left panel), and frequencies of Tfh cells (defined as liveB220^−^CD3e^+^CD4^+^CD44^+^PD-1^+^CXCR5^+^ cells) in cmLN are shown (B, left panel) (pooled from three mice per group). Cumulative data from three individual experiments are summarized (A and B, right panel). The data are presented as the means ± SEM (*n* = 3). Statistical significance was determined using ordinary one-way ANOVA followed by Tukey’s multiple comparisons test. *P < 0.05, **P < 0.01, and ***P < 0.001.

We hypothesized that increased migration of antigen-carrying DCs into the cmLNs, in conjunction with their activation, would enhance the initial stages of adaptive immunities to DS. Therefore, we assessed the quantity of germinal center (GC) B cells in the NALT and cmLNs, as well as T follicular helper (Tfh) cells in the cmLNs, at 12 days post-immunization. The gating strategy is shown in Fig. S2B and C. Mice immunized with rDS plus rLF, rDS-F, and rDS-F plus rLF exhibited 3.5-, 2.7-, and 3.6-fold higher frequencies of DS-specific GC B cells in the NALT, respectively, compared to those mice immunized with rDS alone (upper panel of Fig. 4A). The fold increases of DS-specific GC B cell frequencies in the cmLNs of mice immunized with rDS plus rLF, rDS-F, and rDS-F plus rLF were 7.1-, 7.5-, and 26.4-fold, respectively, compared to those mice immunized with rDS alone (lower panel of Fig. 4A). Regarding Tfh cell responses, the frequency of Tfh cells in the cmLN of mice immunized with rDS or rDS-F alone was similar to that of the mice immunized with PBS. However, mice that received rDS plus rLF and rDS-F plus rLF exhibited 1.7- and 1.9-fold higher frequencies of Tfh cells in the cmLNs, respectively, compared to the mice that received rDS alone (Fig. 4B). Therefore, immunization with rDS-F plus rLF is superior for inducing both GC and Tfh cell responses in the mucosa and nearby lymph nodes.

### Intranasal vaccination of rDS-F plus rLF enhances persistent systemic and mucosal antibody responses

**Fig 5:**
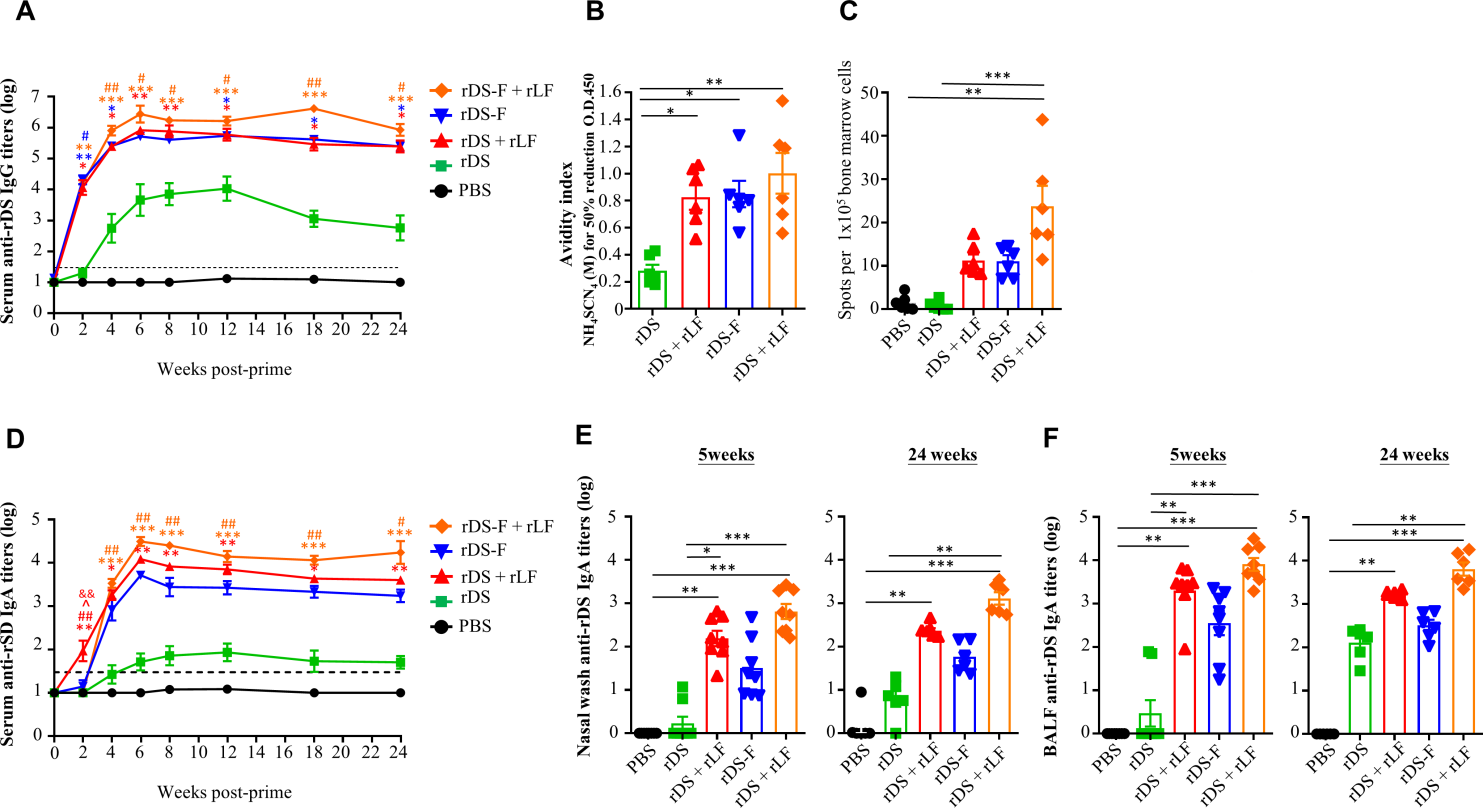
Antibody responses induced by intranasal vaccination of rDS-F plus rLF. Groups of C57BL/6JNarl mice (*n* = 6 or 8/group) were intranasally immunized three times with 20 µg of rDS, rDS plus 10 µg rLF, 20 µg rDS-F, or rDS-F plus 10 µg rLF at 2-week intervals. Mice immunized with PBS alone (without antigens) served as controls. Sera were collected at the indicated time points. Titers of rDS-specific IgG (**A**) and IgA (**D**) in sera were assessed by ELISA. The dashed line indicates the initial fold dilution of serum samples. Data represent the mean ± SE of the mean. Statistical significance was determined using the Kruskal-Wallis test with Dunn’s multiple comparison test. *P < 0.05; **P < 0.01; and ***P < 0.001 vs PBS. #*P* < 0.05 and ##*P* < 0.01 vs rOVA. (**B**) Antibody avidity profiles against rDS were examined by ELISA at 6 weeks after the first immunization. (**C**) rDS-specific antibody-secreting cells in bone marrow were evaluated using ELISpot at 24 weeks after the first immunization. Samples of nasal wash (NW) (**E**) and bronchoalveolar lavage fluid (BALF) (**F**) were collected from mice at 5 or 24 weeks after the first immunization. Reactivity of rDS-specific IgA antibody titers in NW and BALF was assessed by ELISA. Data represent the mean ± SEM of the mean. Statistical significance was determined using the Kruskal-Wallis test with Dunn’s multiple comparison test. *P < 0.05; **P < 0.01; and ***P < 0.001. The dashed line indicates the initial fold dilution of serum samples.

We next evaluated the systemic and mucosal antibody responses. C57BL/6JNarl mice were randomly assigned to groups and received intranasal administrations of 20 µg of rDS with or without 10 µg of rLF and 20 µg of rDS-F with or without 10 µg of rLF, three times at 2-week intervals. PBS-treated (without antigen) mice were used as the control group. As shown in Fig. 5A, anti-DS IgG antibodies were rapidly generated in mice 2 weeks after a single immunization dose of rDS plus rLF, rDS-F, or rDS-F plus rLF. With subsequent boosts, the anti-DS IgG antibody titers in the sera of these three groups were maintained for at least 24 weeks after initial priming without substantial waning. The anti-DS IgG antibody titers in mice immunized with rDS alone were lower than those in the other immunization groups. Furthermore, the antibody avidity profiles of serum samples collected from different groups at 6 weeks were examined. The avidity index of mice immunized with rDS alone was 0.28. Interestingly, the avidity indexes of mice immunized with rDS plus rLF, rDS-F, and rDS-F plus rLF were increased to 0.83, 0.85, and 1.00, respectively (Fig. 5B). These results support the idea that both rDS-F and adding rLF in the antigen not only enhance anti-DS IgG antibody titers but also increase the avidity of antibodies. Next, we evaluated the frequency of antibody-secreting cells (ASCs) using enzyme-linked immunospot (ELISpot) analysis in the bone marrow at 24 weeks after initial priming. Again, mice immunized with rDS-F plus rLF retained a higher population of ASCs (Fig. 5C).

In terms of IgA antibody responses, the anti-DS IgA antibody titers in the sera had profiles similar to those of anti-DS IgG antibody titers in the sera. The anti-DS IgA antibody titers were maintained for at least 24 weeks after initial priming and were higher in mice immunized with rDS plus rLF, rDS-F, and rDS-F plus rLF than in mice immunized with rDS alone (Fig. 5D). Local mucosal responses can be assessed by measuring the levels of secretory IgA (sIgA) in the nasal wash (NW) and bronchoalveolar lavage fluid (BALF). Therefore, we monitored sIgA titers in NW (Fig. 5E) and BALF (Fig. 5F) at 5 and 24 weeks after the initial priming. Immunization with rDS plus rLF, rDS-F, and rDS-F plus rLF induced sustained mucosal anti-DS IgA responses in NW and BALF. In contrast, immunization with rDS alone elicited only weak to undetectable IgA responses. These results suggest that both rDS-F and the addition of rLF to the antigen can promote mucosal IgA antibody responses. All of the results indicate that the combination of rDS-F and rLF is the most effective formula for inducing superior systemic and mucosal responses.

### Intranasal vaccination of rDS-F plus rLF promotes robust T-cell responses

**Fig 6:**
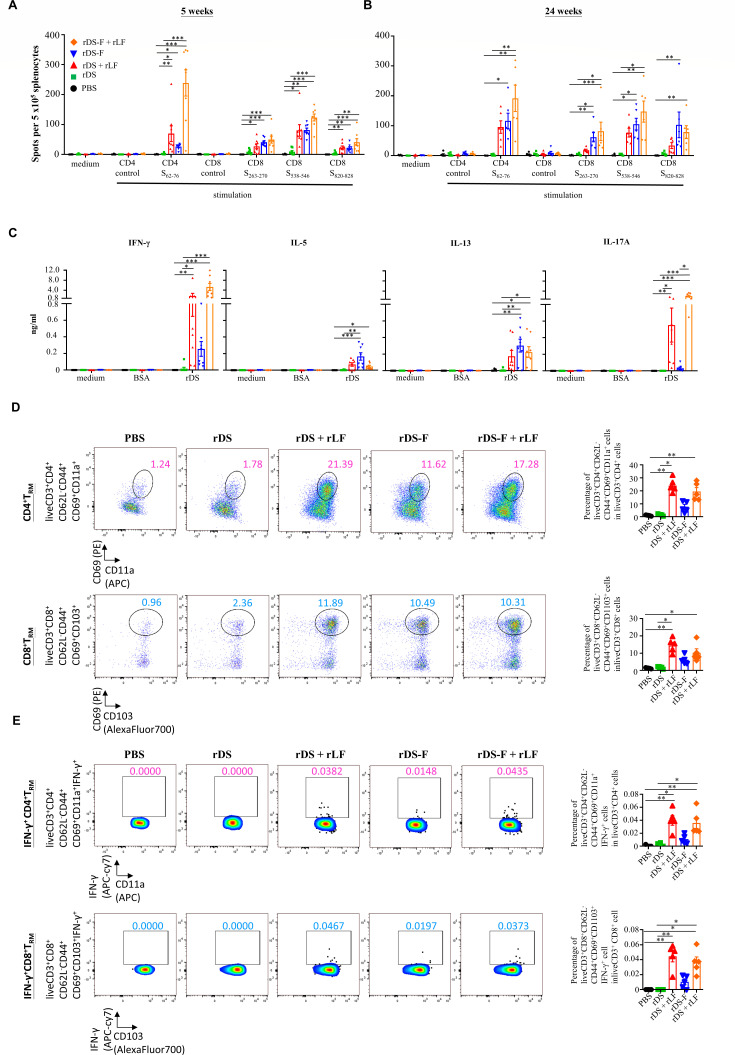
T-cell responses and cytokine production profiles induced by intranasal vaccination of rDS plus rLF. Groups of C57BL/6JNarl mice (*n* = 5 or 8/group) were intranasally immunized three times with 20 µg of DS rDS plus 10 µg rLF, 20 µg rDS-F, or rDS-F plus 10 µg rLF at 2-week intervals. Mice immunized with PBS alone (without antigens) served as controls. Splenocytes were harvested at 5 (**A**) or 24 weeks (**B**) after the first immunization. Cells were cultured and stimulated with SARS-CoV-2 spike protein-derived peptides or control peptides for 3 days in an anti-INF-γ-coated 96-well ELISpot plate. IFN-γ-producing cells are expressed as spot-forming units per 5 × 10^5^ cells. Spleens and lungs were harvested at 5 weeks after the first immunization. (**C**) Splenocytes were cultured and stimulated with rDS for 3 days. Stimulation with bovine serum albumin or medium alone served as controls. Cytokine profiles in the supernatants were evaluated by ELISA. (**D and E**) The pneumocytes were cultured and stimulated with rDS for 18 hours and then analyzed by flow cytometry. Representative flow cytometry gating and percentage of CD4^+^ and CD8^+^ T_RM_ cells (D, left panel) and percentage of IFN-γ-producing CD4^+^ and CD8^+^ T_RM_ cells (E, left panel) in total CD4^+^ and CD8^+^ T cells. Data from individual mice are summarized (D and E, right panel). Statistical significance was determined using the Kruskal-Wallis test with Dunn’s multiple comparison test (**A–C**) or ordinary one-way ANOVA followed by Tukey’s multiple comparisons test (**D and E**). *P < 0.05; **P < 0.01; and ***P < 0.001.

To assess the T-cell responses, we used the same vaccination regimen and schedule as described in the above section of antibody studies. One week after the last immunization, the frequencies of IFN-γ-producing cells in the spleens were evaluated by ELISpot. The frequencies of IFN-γ-producing cells in the splenocytes were at background levels when there was no stimulation (medium) or stimulation with control peptides for all the groups. Mice immunized with rDS alone induced only weak or background levels of IFN-γ-producing cells after stimulating with S_62-76_ peptide (a CD4-specific epitope of SARS-CoV-2 spike protein) (38) or S_263-270_, S_538-546_, and S_820-828_ (all are CD8-specific epitope of SARS-CoV-2 spike protein) (38) (Fig. 6A). Remarkably, mice immunized with rDS plus rLF, rDS-F, and rDS-F plus rLF all induced substantial levels of IFN-γ-producing cells after stimulating with the CD4 epitope (S_62-76_) or CD8 epitopes (S_263-270_, S_538-546_, and S_820-828_) and maintained at least 24 weeks after the priming (Fig. 6B). These findings provide evidence that either rDS-F alone or antigen formulation with rLF can stimulate both CD4 and CD8 T-cell responses.

To further evaluate the T helper cell profiles, we examined the levels of IFN-γ (a Th1-associated cytokine), IL-5 and IL-13 (Th2-associated cytokines), and IL-17A (a Th17-associated cytokine) produced by the splenocytes of vaccinated mice. As shown in Fig. 6C, supernatants obtained from all splenocytes secreted low or barely detectable levels of these cytokines without stimulation (medium) or stimulation with a control protein (bovine serum albumin, BSA). Consistent with the ELISpot results, mice immunized with rDS alone induced only weak or background levels of these cytokines after stimulation with rDS. In contrast, mice immunized with rDS plus rLF, rDS-F, and rDS-F plus rLF all produced substantial levels of IFN-γ, IL-5, IL-13, and IL-17A. Furthermore, we observed that mice immunized with rDS-F plus rLF produced more IFN-γ and IL-17A than mice immunized with other formulations. These findings suggest that rDS-F plus rLF induces Th1/Th17-biased responses.

Next, we assessed the induction of lung tissue-resident memory T (T_RM_) cells by different vaccination schemes. The gating strategy is shown in Fig. S3. Both lung CD4^+^ and CD8^+^ T_RM_ cells in mice immunized with rDS alone were equivalent to those in mice immunized with PBS. Mice immunized with rDS-F alone exhibited elevated CD4^+^ and CD8^+^ T_RM_ cells in the lung compared to those in mice immunized with PBS or rDS alone. Significantly, adding rLF to rDS or rDS-F further increased CD4^+^ and CD8^+^ T_RM_ cells in the lung (Fig. 6D). Antigen-specific CD4^+^ and CD8^+^ T_RM_ cells were identified by *ex vivo* restimulation with rDS, followed by intracellular staining of IFN-γ. The highest percentages of IFN-γ-producing CD4^+^ and CD8^+^ T_RM_ cells were detected in the lungs of mice that received rDS or rDS-F vaccines supplemented with rLF (Fig. 6E). These results indicate that formulating antigens with rLF is an effective strategy for inducing antigen-specific CD4^+^ and CD8^+^ T_RM_ cells in the lung.

### Combination of rLF with rDS or rDS-F induces broadly neutralizing antibody responses against SARS-CoV-2 variants

**Fig 7:**
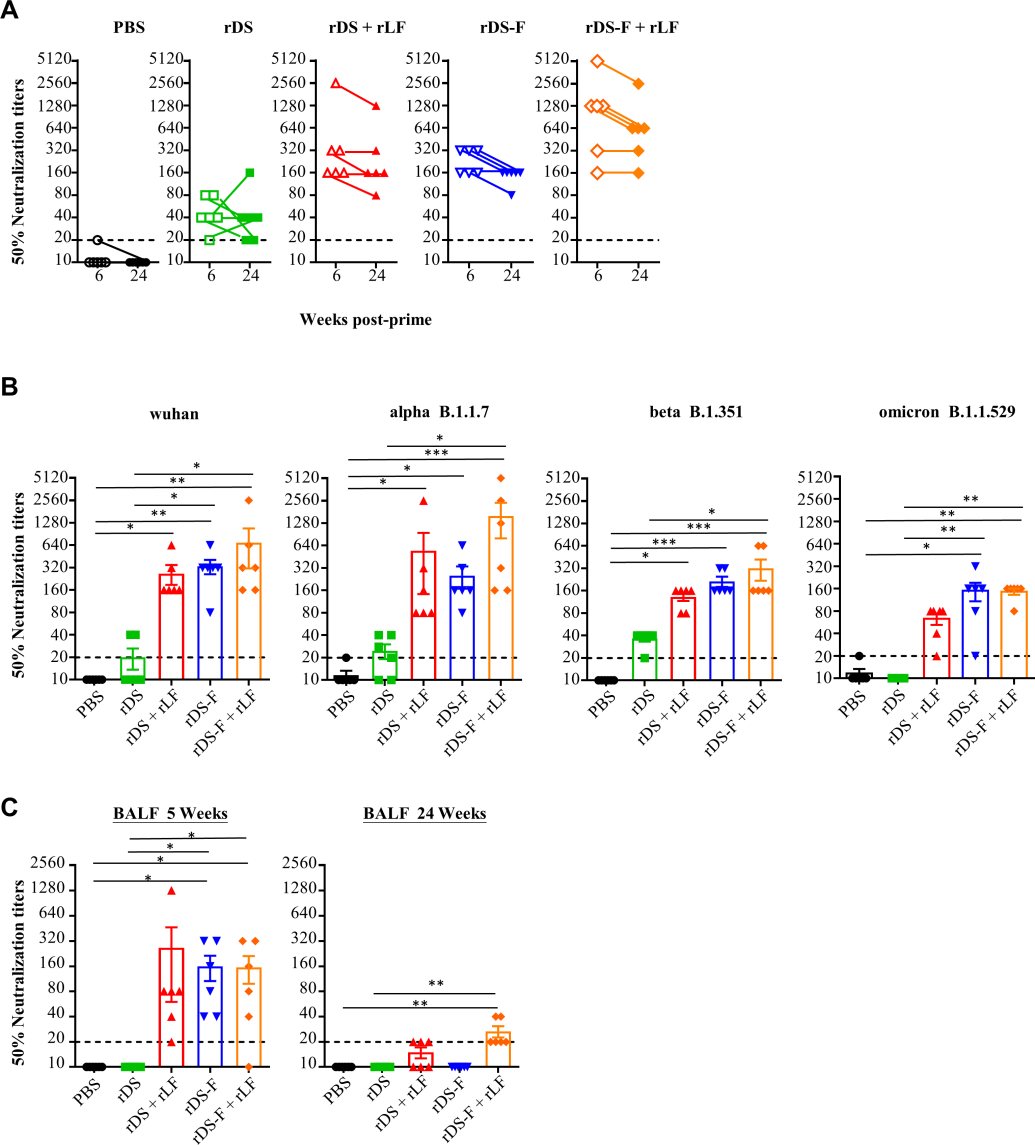
Intranasal vaccination with rDS-F plus rLF enhanced systemic and mucosal neutralizing antibody against SARS-CoV-2 and its variants. Groups of C57BL/6JNarl mice (*n* = 6 or 8/group) were intranasally immunized three times with 20 µg of rDS, rDS plus 10 µg rLF, 20 µg rDS-F, or rDS-F plus 10 µg rLF at 2-week intervals. Mice immunized with PBS alone (without antigens) served as controls. Sera and BALF were collected at the indicated time points. The neutralizing antibody titers (50% neutralization titers) against the Delta strain of SARS-CoV-2 (**A**), Wuhan, and other variants (Alpha, Beta, and Omicron) (**B**) were measured in the serum. (**C**) The neutralizing antibody titers against the Delta strain of SARS-CoV-2 were measured in the BALF. Data represent the mean ± SE of the mean. Statistical significance was determined using the Kruskal-Wallis test with Dunn’s multiple comparison test. *P < 0.05; **P < 0.01; and ***P < 0.001. The dashed line indicates the initial fold dilution of serum samples.

To evaluate the neutralizing ability against SARS-CoV-2, samples of serum and BALF collected in the antibody studies described above were subjected to neutralization assays. Mice immunized with rDS alone elicited a detectable neutralizing antibody response in their sera against Delta SARS-CoV-2 that persisted for 24 weeks after priming. Remarkably, neutralizing antibody titers were further increased in mice immunized with rDS-F alone or in combination of rDS or rDS-F with rLF. Notably, neutralizing titers remained stable or only slightly decreased for at least 24 weeks after priming (Fig. 7A).

Furthermore, we evaluated the neutralizing capacity of sera collected at 6 weeks after priming against Wuhan, Alpha, Beta, and Omicron SARS-CoV-2. Substantial neutralizing antibody titers were detected in mice immunized with rDS plus rLF, rDS-F, or rDS-F plus rLF (Fig. 7B). These results suggest that either rDS-F alone or mixing rLF with rDS or rDS-F can stimulate broadly neutralizing antibody responses against homologous Delta SARS-CoV-2 and heterologous variants.

BALF samples collected at 5 or 24 weeks after priming were assessed for neutralizing capacity against Delta SARS-CoV-2 (Fig. 7C). There were no detectable neutralizing abilities in the mice immunized with PBS or rDS alone. In contrast, mice immunized with rDS-F alone or mixing rLF with rDS or rDS-F induced neutralizing abilities in the BALF. The neutralizing capacities were waned in the BALF. Noteworthy, at 24 weeks after priming, the neutralizing capacities were still present in the mice immunized with rDS-F plus rLF. These results suggest that rDS-F plus rLF elicited a superior neutralizing capacity in systemic and mucosa sites.

### Combining rLF with rDS or rDS-F elicits superior protective responses against SARS-CoV-2 variants

**Fig 8:**
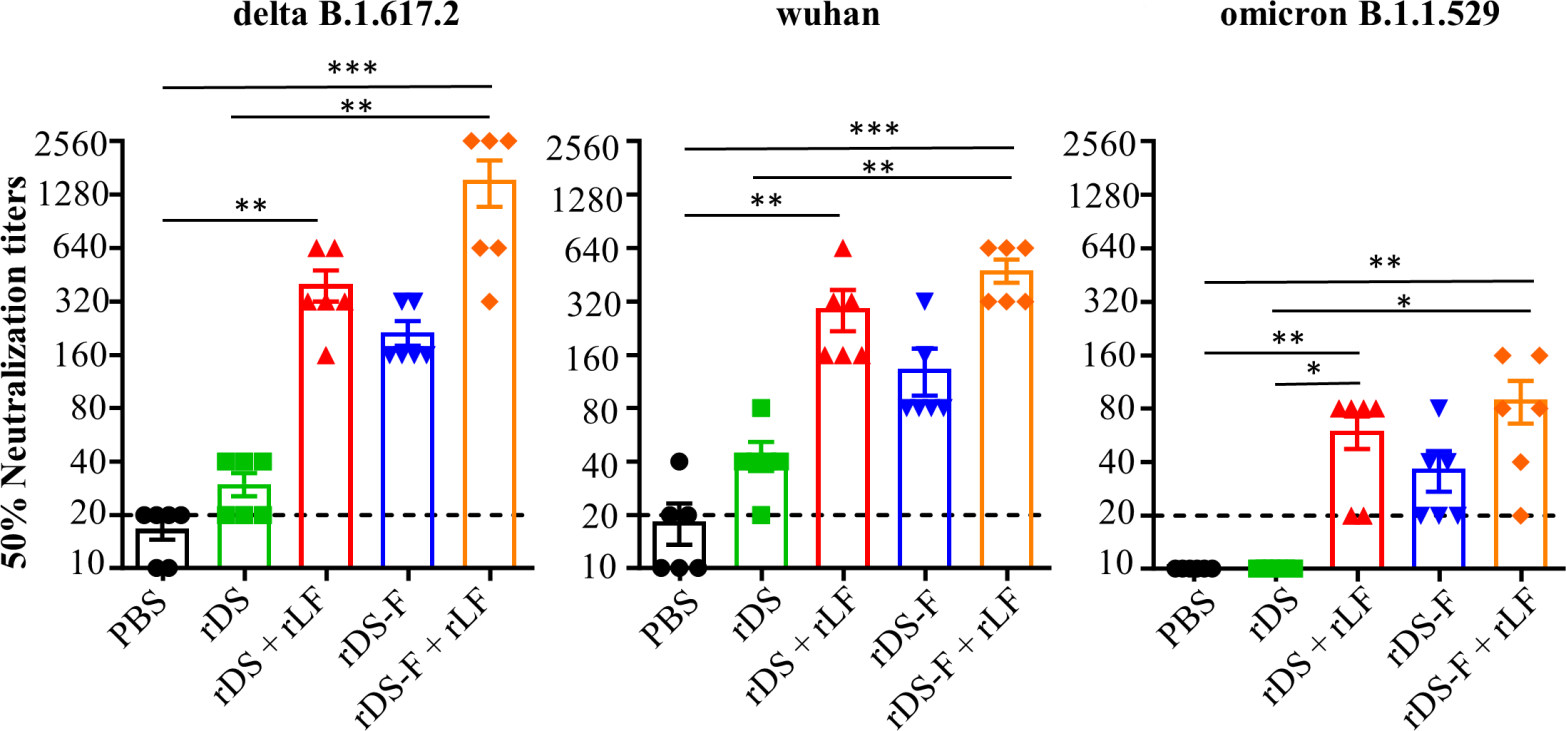
Induction of neutralizing antibodies against SARS-CoV-2 variants in hamsters through intranasal vaccination with rDS-F plus rLF. Groups of hamsters (*n* = 6/group) were intranasally immunized three times with 20 µg of rDS, rDS plus 10 µg rLF, 20 µg of rDS-F, rDS-F plus 10 µg rLF, or PBS at 2-week intervals. Hamsters immunized with PBS alone (without antigens) served as controls. Sera were collected at 2 weeks after the last immunization. The neutralizing antibody titers (50% neutralization titers) against the Delta, Wuhan, and Omicron strains of SARS-CoV-2 were measured in the serum. Data represent the mean ± SE of the mean. Statistical significance was determined using the Kruskal-Wallis test with Dunn’s multiple comparison test. *P < 0.05; **P < 0.01; and ***P < 0.001. The dashed line indicates the initial fold dilution of serum samples.

**Fig 9:**
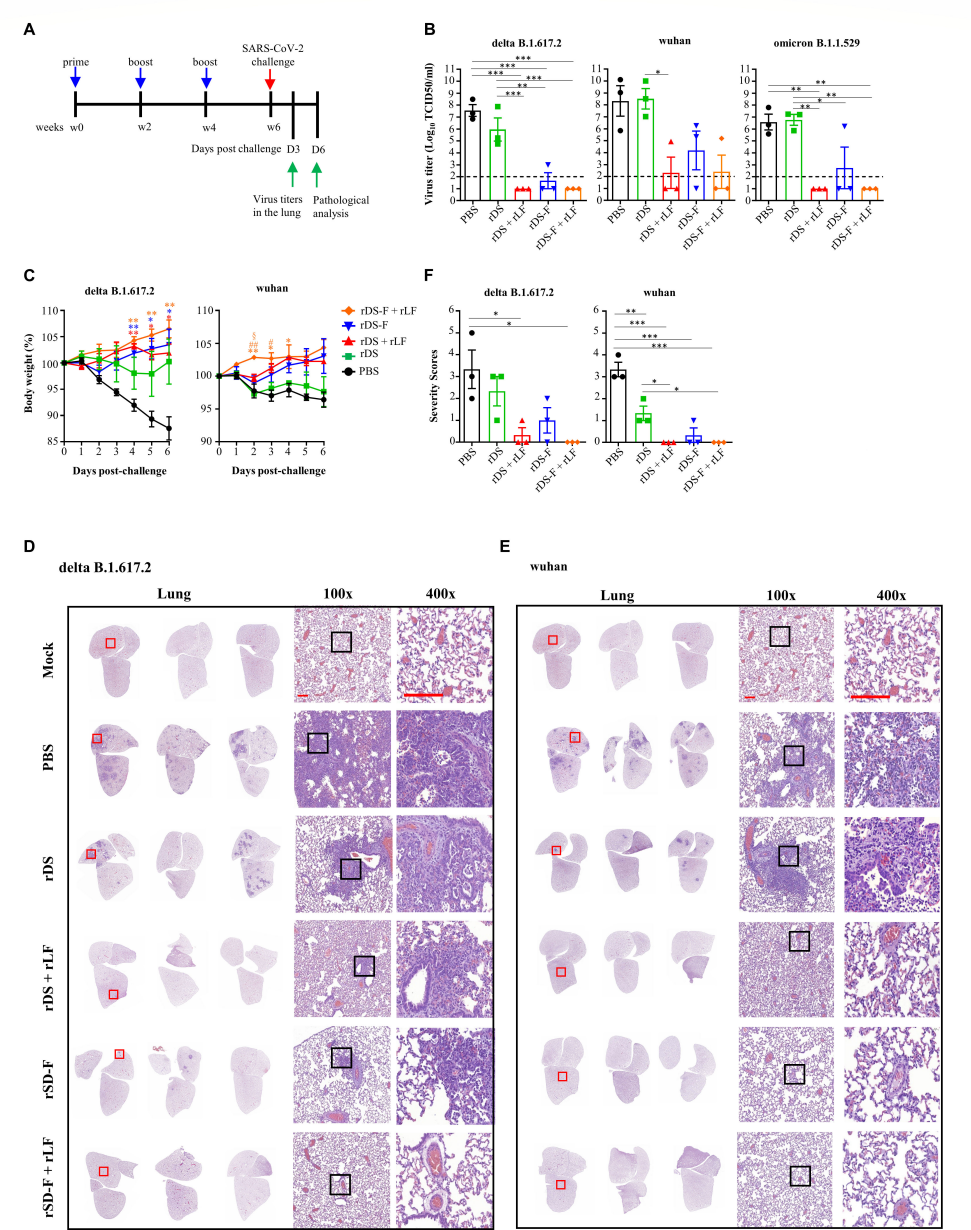
Effectiveness of intranasal vaccination in protecting hamsters against infection by SARS-CoV-2 and its variants. (**A**) Groups of hamsters (*n* = 3/group) were intranasally immunized three times with 20 µg of rDS, rDS plus 10 µg rLF, 20 µg of rDS-F, rDS-F plus 10 µg rLF, or PBS at 2-week intervals. All animals were intranasally challenged with SARS-CoV-2 (1 × 10^4^ TCID_50_/hamster for Wuhan, Delta, and Omicron) at 6 weeks after the first immunization. (**B**) The viral load in the lungs was assessed on day 3 following the virus challenge. The dashed line indicates the limit of detection. (**C**) Body weight changes (%) relative to the day of the viral challenge are plotted. Statistical significance was determined using the ordinary one-way ANOVA followed by Tukey’s multiple comparisons test. *P < 0.05 and **P < 0.01 vs PBS; #*P* < 0.05 and ##*P* < 0.01 vs rDS; §*P* < 0.05 vs rDS-F. (**D and E**) Lung sections were analyzed at day 6 post-infection using H&E staining. Panels show complete sections from three hamsters per group, with magnified images in the middle and right panels. Scale bars (red line) indicate 100 µm. The mock tissue sections in panels D and E are identical for reference purposes. The first image of the mock tissue section is adapted from our previous study (40). (**F**) Pathological severity scores were evaluated according to the percentage of inflammation area for each section from each animal using the following scoring system: 0, no pathological change; 1, affected area ≤ 10%; 2, affected area 10%–30%; 3, affected area 30%–50%; and 4, affected area ≥ 50%. Statistical significance was determined using the ordinary one-way ANOVA followed by Tukey’s multiple comparisons test. *P < 0.05 and **P < 0.01.

We further evaluated the protective effects against SARS-CoV-2 *in vivo* after immunization. Hamsters were randomly assigned to different groups and received intranasal administrations of either 20 µg of rDS with or without 10 µg of rLF or 20 µg of rDS-F with or without 10 µg of rLF, three times at 2-week intervals. Administration of PBS was used as a placebo control in parallel. Serum samples collected 6 weeks post-priming were evaluated for neutralizing capacity against SARS-CoV-2 variants Delta, Wuhan, and Omicron (Fig. 8). Consistent with the findings in mice, substantial neutralizing antibody titers were detected in hamsters immunized with rDS plus rLF, rDS-F, or rDS-F plus rLF. These results suggest that either rDS-F alone or mixing rLF with rDS or rDS-F can induce broadly neutralizing antibody responses against homologous Delta SARS-CoV-2 and heterologous variants. Two weeks after the last immunization, hamsters were challenged with SARS-CoV-2 via the intranasal route. Each group of hamsters was divided into two subgroups: one subgroup was sacrificed on day 3 post-infection to evaluate the viral loads in their lungs, while the other subgroup was monitored for changes in body weight over a 6-day period and then examined for lung histopathology, except for the Omicron challenge studies. This is because hamsters infected with the Omicron variant of SARS-CoV-2 did not experience significant body weight loss or severe lung pathology (39). The experimental scheme is shown in Fig. 9A.

High SARS-CoV-2 virus titers were present in both the administration of PBS and rDS alone groups (Fig. 9B). Virus titers were reduced in the group administered with rDS-F. No detectable virus was found in two, one, and two out of three hamsters challenged with Delta, ancestral, and Omicron, respectively. When rLF is combined with rDS or rDS-F, virus replication in the lungs was further suppressed. Upon Delta and Omicron SARS-CoV-2 challenge, no detectable virus was found in all three hamsters. Two out of three hamsters did not show infected virus when challenged with Wuhan SARS-CoV-2. Compared to the PBS group, there was no SARS-CoV-2-induced body weight loss observed in hamsters that were administered with rDS plus rLF, rDS-F, or rDS-Plus rLF groups (Fig. 9C). For lung histopathological evaluation, severe lung inflammation was observed in the PBS group after challenged with Delta and Wuhan SARS-CoV-2 (Fig. 9D and E), which led to high pathological severity scores (Fig. 9F). Fewer lung pathological changes were observed in hamsters that were administered with rDS-F alone after Delta or Wuhan SARS-CoV-2 challenge. Consistent with the viral loads in the lungs, hamsters that were administered with rDS plus rLF or rDS-F plus rLF showed no or few lung pathological changes, which were similar to those of no infection, after the Delta or Wuhan SARS-CoV-2 challenge. These results suggest that the combination of rLF with rDS or rDS-F elicits superior protective responses against SARS-CoV-2 variants in hamsters.

## DISCUSSION

The COVID-19 pandemic has had a profound impact on the global community, affecting social, economic, and healthcare aspects, and continues to do so. Currently, efforts are still underway to develop a vaccine capable of generating mucosal immunity at the entry site of SARS-CoV-2 infections and providing extensive protection against various variants. In our previous studies, we established a targeted antigen delivery system by fusion antigen with FLIPr (22). Our results demonstrate that FLIPr is a competent vehicle for effectively delivering antigens to DCs, thereby triggering robust antigen-specific immune responses. Recently, we have also discovered that rLF not only serves as a potential vaccine candidate, inducing potent antibody responses to overcome FLIPr-mediated inhibition of phagocytosis, but also functions as a powerful adjuvant. This occurs through the simple mixing of rLF with the antigen (37). Therefore, we hypothesized that the immunostimulatory properties of rLF can further activate DCs loaded with rDS-F, thereby synergizing immune responses. In this study, we employ these two technologies and demonstrate that combining rDS-F and rLF is an effective vaccine candidate for inducing robust immune responses and protecting against multiple variants of SARS-CoV-2 infections.

DCs, the most potent professional APCs, capture and process antigens in peripheral tissues, migrate to lymphoid organs, and initiate the differentiation of naive T cells (CD4^+^ or CD8^+^ T cells) into effector cells (helper or cytotoxic T cells), while also regulating humoral immune responses (41–43). Therefore, delivering antigens efficiently to DCs after vaccination is crucial for triggering a robust adaptive immune response. In addition, delivery of antigens to DCs under steady-state conditions results in a transient expansion of T-cell clones, which does not persist in the long term and leads to tolerance. In contrast, targeting antigens to DCs along with DC activation agents promotes the proliferation of T-cell clones, leading to antigen-specific immunity (44–47). Therefore, the delivery of antigens to DCs and the simultaneous stimulation of DC activation are crucial for triggering a robust adaptive immune response. In line with this notion, we demonstrate that intranasal administration of rDS-F plus rLF increases the amount of antigen attached to the nasal mucosa, promotes antigen penetrated into the tissues (Fig. 2), and increases antigen captured by DCs (Fig. 3A). Importantly, these antigen-loaded DCs express high levels of CD40, CD80, and CD86 (Fig. 3B), which indicate these DCs are in activated-state and favor to induce antigen-specific immune responses.

Both NALT and lymph nodes play a critical role in initiating and coordinating mucosal antigen-specific immune responses (48, 49). Consistent with the efficient delivery of antigen to DCs following intranasal immunization with rDS-F plus rLF (Fig. 3), we observed a significant increase in total Tfh cells in the cmLN and antigen-specific GC B cells in NALT and cmLN (Fig. 4). These findings were associated with enhanced systemic IgG and IgA responses, as well as mucosal antibody responses (Fig. 5). Besides SARS-CoV-2, several pathogens like influenza, rotavirus, RSV, and cholera are believed to necessitate a combination of mucosal IgA and serum IgG antibodies to provide the best protection (9, 50–54). Thus, combining the antigen-FLIPr fusion protein with rLF is a promising strategy for developing vaccines against diverse pathogens.

Mucosal immune response serves as the first line of defense against most pathogen infections. Among them, mucosal IgA facilitates the removal of pathogenic microorganisms through a process that involves impeding their access to epithelial receptors, entrapping them within the mucus layer, and promoting their clearance through peristaltic and mucociliary actions (55). Clinical data shows that SARS-COV-2 infection early virus-specific body fluid response is mainly IgA antibody rather than IgG. Especially in the lungs, IgA contributes to SARS-CoV-2 neutralization to a greater extent than IgG (56). Recent clinical studies provide evidence that high concentrations of nasal mucosal SARS-CoV-2 WT spike IgA can substantially protect against Omicron infection (57). Several studies support the importance of antigen presentation by the mucosal route for mucosal protection and highlight the direction of future next-generation vaccine development (58–61). Here, we demonstrate that delivery through nasal immunization with rDS plus rLF, rDS-F, or rDSF plus rLF results in a significant increase in neutralizing IgA against SARS-CoV-2 in the local mucosa (Fig. 5E and F). Especially in the rDS-F plus rLF group, the neutralized antibodies in mucosa at least lasted for 24 weeks (Fig. 7C). This suggests the synergistic effect of combining FLIPr-fused antigens adjuvanted with rLF in inducing mucosal immune responses.

Both CD4^+^ and CD 8^+^ T cells were critical to clear the SARS-CoV-2 during primary infection in mice (62). The significantly higher levels of SARS-CoV-2-specific CD4 T cells have been considered to have a strong association with the increase in convalescent neutralizing antibodies (63, 64). Relatively, the SARS-CoV-2-specific CD8^+^ T-cell responses may be related to the severity of the acute phase of the disease (65, 66). The weak CD8^+^ T-cell responses could impact the pathogenesis of acute COVID-19 despite the presence of high antibody titers (67). Furthermore, several studies have indicated the significant contribution of CD8^+^ T cells in vaccine protection against SARS-CoV-2 infection, which may also prevent reinfection with different variants (19, 68, 69). Our study revealed that immunization with rDS plus rLF, rDS-F, or rDS-F plus rLF can effectively elicit a specific CD4^+^ and CD8^+^ T-cell response against SARS-CoV-2 (Fig. 6). It is worth noting that CD8-specific epitopes are highly conserved across different variants of SARS-CoV-2 (Table S1). This may explain why, despite the neutralizing antibody against the Omicron strain being over eight times lower than that against the Delta strain (Fig. 7 and 8), a considerable degree of protection against Omicron was still maintained during infection (Fig. 9).

Antibodies generated by long-lived plasma cells, memory T, and B cells are the major immune responses providing long-term protection for the host against the reinfection by the same or similar pathogens. Mice that were immunized with rDS-F and rLF maintained a substantial population of DS-specific antibody-producing cells in the bone marrow even after 24 weeks of immunization. Importantly, IgG and IgA were persistent in the blood and mucosa (Fig. 5). These results provide the foundation for long-term protection. SARS-CoV-2 continues to mutate, changing its surface antigens to escape antibody-mediated neutralization (7). Thus, memory T cells, in particular lung T_RM_, are thought to be another crucial immunity in providing effective protection against the spread of viruses and the development of severe diseases (70–73). In this study, mixing the antigen with rLF can increase IFN-γ producing CD4^+^ and CD8^+^ T_RM_ in the lung (Fig. 6D and E), which correlates with better protection against SARS-CoV-2 challenge (Fig. 9). However, having more T_RM_ does not always mean better immunity (74, 75). In elderly individuals who have recovered from COVID-19, the number of CD8^+^ T_RM_ cells in the respiratory tract seems to be positively associated with post-COVID-19 lung sequelae and dysfunction (76, 77). Further research is necessary to understand the mechanisms that regulate the function of T_RM_ cells, in order to harness their protective properties and minimize their pathogenic potential during the development of vaccines.

Safety is a crucial concern for any vaccine, especially when considering that FLIPr originates from *Staphylococcus aureus* and can inhibit IgG-mediated effector functions (78). Previously, we assessed serum biochemical, hematological, and histopathologic parameters after three repeated doses of rLF administration. There were no significant differences between the mice administered with PBS or rLF. In addition, rLF also induced functional antibodies that effectively blocked FLIPr-mediated inhibition of phagocytosis. These results suggest that rLF is a safe adjuvant with additional benefits in abolishing FLIPr-mediated immunosuppressive effects (37). The presence of anti-FLIPr antibodies may raise another consideration, which could potentially compromise subsequent immune responses induced by the antigen-FLIPr fusion protein or the adjuvant effects of rLF. However, these concerns have been addressed in our previous studies, demonstrating that pre-existing antibodies against FLIPr in mice do not diminish the subsequent immune response induced by antigen-FLIPr fusion proteins (79) and do not negate rLF adjuvant effects (37). Altogether, the formulation of the antigen-FLIPr fusion protein with rLF is a potent strategy for vaccine development.

A successful vaccine must be able to induce both high-quality and quantity immune responses as well as long-lasting memory responses. The findings of this study demonstrate that the combination of rDS-F and rLF elicits durable protective antibody and T-cell responses, both systemically and locally in the mucosa. In conclusion, our results support that rDS-F plus rLF is a promising vaccine candidate against multiple SARS-CoV-2 variants. Additionally, antigen fused with FLIPr and adjuvanted with rLF represents an effective strategy for developing vaccines.

## MATERIALS AND METHODS

### Production of rDS, rDS-F, and rLF

The production and purification of rLF were described previously (37) (Supplementary Methods). The generation process of pDS was mostly similar to previous studies (80), albeit with some modifications. A DNA fragment was designed to encode the Delta (B.1.617.2) strain of the SARS-CoV-2 spike protein (100% match to accession number: 7V7N_A). The fragment contains a non-functional furin cleavage site (R680G, R681S, and R683S) and two stabilizing prolines (K984P and V985P) in the hinge loop. Additionally, the transmembrane domain and the C-terminal intracellular tail were replaced by a trimerization domain IZN4, followed by eight residues of histidine at the carboxy terminus for purification. DNA sequence of the S variant was codon-optimized for Homo sapiens, fully synthesized, and cloned into the pcDNA3.1(+) (Novagen, Madison, WI, USA) plasmid vector by GenScript (Piscataway, NJ, USA) to generate pDS.

The pcDNA3.1(+)-FLIPr plasmid first needed to be constructed before the generation of pDS-F. To generate pcDNA3.1(+)-FLIPr, a forward primer, 5′-CCGCTCGAGTTCTTTAGC TACGAGTGGAAGG-3′ (the XhoI site is underlined), a reverse primer, 5′-TGCTCTAGATTAGT GATGATGGTGGTGGTGGTGG-3′ (the XbaI site is underlined), and pOVA-FLIPr (as template) (21) were used to clone the FLIPr sequence into the XhoI and XbaI sites of the plasmid pcDNA3.1(+). The forward primer, 5′-CTAGCTAGCATGTTTGTCTTC CTGGTC-3′ (NheI site is underlined) combined with reverse primer, 5′- CCGCTCGAGCCTCCC TCCAGTTCTGTTTCC-3′ (XhoI site is underlined) were used to amplify the synthetic DNA of DS. The PCR product was then cloned into the NheI and XhoI sites of the pcDNA3.1(+)-FLIPr to produce the plasmid pDS-F.

The production of rDS and rDS-F was achieved using the ExpiCHO Expression System Kit (ThermoFisher Scientific, Carlsbad, CA, USA) following the manufacturer’s instructions. Briefly, pDS and pDS-F were first transfected into ExpiCHO cells (cultured in ExpiCHO Expression Medium) using the ExpiFectamine CHO Reagent. The cells were then incubated in a 37°C incubator with a humidified atmosphere of 8% CO_2_ in air on an orbital shaker. One day after transfection, ExpiFectamine CHO Enhancer and ExpiCHO Feed were added to the flask, and then the flask was transferred to a 32°C incubator with a humidified atmosphere of 5% CO_2_ in air and shaken. At the time of protein harvest (10 days post-transfection for rDS expression and 5 days post-transfection for rDS-F expression), the culture medium containing rDS or rDS-F was centrifuged at 46,500 × *g* for 30 min at 4°C; later, the supernatant was filtered through a 0.22-µm filter and dialyzed with equilibration buffer (50 mM Tris-HCl, 150 mM NaCl, and 20 mM imidazole, pH 8.9 for rDS, and 20 mM Tris, 50 mM sucrose, 500 mM NaCl, and 10% glycerol, pH 8.5 for rDS-F).

To purify rDS or rDS-F, the supernatant after being dialyzed was loaded onto immobilized metal affinity chromatography columns (Bio-Rad, Hercules, CA, USA) (2.5 cm i.d. × 10.0 cm) containing Ni-NTA resin (Qiagen, San Diego, CA, USA) to bind rDS or rDS-F. The column was washed with a 20-fold column volume of equilibration buffer additionally containing 20 mM imidazole and then rDS or rDS-F was eluted with equilibration buffer with 500 mM imidazole. Finally, the eluted rDS or rDS-F was dialyzed with dialysis buffer (20 mM sodium phosphate, pH = 8.0 for rDS, and 20 mM Tris and 200 mM NaCl, pH 8.5 for rDS-F) three times for at least 6 hours each time. The rDS and rDS-F were lyophilized and stored at −20°C. The fractions from each step were analyzed using SDS-PAGE and immunoblotted with anti-His tag antibodies.

### Animals

C57BL/6JNarl and Syrian hamsters were obtained from the National Laboratory Animal Breeding and Research Center (Taipei, Taiwan). Mice or hamsters were used between 6 and 8 weeks of age. All the mice and Syrian hamsters were housed at the Laboratory Animal Center of the NHRI.

### Immunofluorescence staining of nasal cavity tissue

For the preparation of nasal cavity samples, C57BL/6JNarl mice were processed as previously described with some modifications (22). Mice were euthanized by controlled administration of carbon dioxide inhalation. Tissue samples were collected and fixed in a 10% neutral buffered formalin solution (Sigma-Aldrich, St. Louis, MO, USA) for 24 hours at room temperature, followed by 10% EDTA (Leica) for approximately 2 weeks for decalcification. The decalcified heads were then dehydrated and embedded in paraffin blocks (Sakura Tissue-Tek TEC 6).

For immunofluorescence staining, the tissue block was cut into 5-µm sections, deparaffinized with multiple passages in xylene, re-hydrated in decreasing concentrations of ethanol, and then rinsed in water. For antigen retrieval, the hydrated slides were immersed in Trilogy (Cell Marque) at 90°C for 20 min. After rinsing with PBS, sections were permeabilized with 0.1% Triton X-100 for 15 min. After three washes with 0.01% Tween/PBS for 5 min, the sections were blocked with 2.5% BSA in PBS for 30 min in a humidiﬁed chamber at room temperature. The antigen in the nasal cavity was detected by staining with mouse anti-SARS-CoV-2 spike protein antibody (Mab3-2) (81) and then labeling with Alexa Fluor 488-conjugated goat anti-mouse Fc antibody (Biolegend, Cat#405319, clone Poly4053) (Biolegend, San Diego, CA, USA). DAPI (Sigma-Aldrich) was used to stain the nucleus. The confocal fluorescent images were acquired using the same settings on a Leica TCS SP5 confocal microscope, followed by image analysis using Leica Application Suite X version 4.3.0.24308. The pixel calculation was performed using ImageJ Ver.1.54d software, and the quantity of S protein deposited in a single field was determined by dividing the total pixel count of the S protein positive area by the total cell area (identified through DAPI positive staining).

### Isolation of NALT, cmLN, and lung single-cell preparation

The isolation of NALT and the preparation of a single NALT cell suspension were performed as described previously (22) (Supplementary Methods). For analyzing cmLN cells or pulmonary cells, cervical and mediastinal lymph nodes or lung tissue were mechanically disrupted and transferred to conical tubes. Lymph nodes and lung tissue were resuspended in 1 mL of RPMI containing 0.4 mg/mL collagenase (Sigma-Aldrich) and incubated for 30 min at 37°C. The lymph nodes and lung tissues were ground and the suspension was collected and passed through a 70 mm cell strainer. The cells were pelleted by centrifugation at 300 × *g* for 5 min.

### Analysis of antigen-carrying dendritic cells in NALT and cmLN

rDS and rDS-F were labeled with an Alexa Fluor 700 labeling kit (Abcam, Cambridge, UK). Groups of C57BL/6JNarl mice were intranasally administered with 20 µg of Alexa Fluor 700-labeled rDS, 20 µg of Alexa Fluor 700-labeled rDS plus 10  µg of rLF, 20  µg of Alexa Fluor 700 labeled-rDS-F, or 20 µg Alexa Fluor 700 labeled-rDS-F plus 10  µg of rLF. At 18 hours after administration, mice were euthanized by controlled administration of carbon dioxide inhalation. Subsequently, a single NALT cell suspension or cmLN cell suspension (pooled from three mice) was prepared as described in the previous sections. Single cells were then incubated with anti-mouse CD16/CD32 antibody (Biolegend, Cat#101335, clone 93) for 30 min on ice to block nonspecific binding on Fc receptors. The Zombie Violet Fixable viability kit (Biolegend, Cat# 423113) was used to evaluate the viability of NALT and cmLN cells by flow cytometry. Lymphocytes were distinguished by staining with PE/Cyanine7-conjugated anti-mouse CD45 antibody (Biolegend, Cat#103114, clone 30-F11). Dendritic cells were distinguished by staining with Alexa Fluor 488-conjugated anti-mouse-MHCII antibody (Biolegend, Cat#107616, clone M5/114.15.2) and PE-conjugated anti-mouse CD11c antibody (Biolegend, Cat#117307, clone N418). Expression levels of costimulatory molecules were evaluated by staining with APC/Cyanine7-conjugated anti-mouse CD40 antibody (Biolegend, Cat#124638, clone 3/23), PerCP/Cyanine5.5 anti-mouse CD80 antibody (Biolegend, Cat#104722, clone 16-10A1), and Brilliant Violet 510-conjugated anti-mouse CD86 antibody (Biolegend, Cat#105040, clone GL-1). The frequency of the antigen-positive (Alexa Fluor 700^+^) DCs and expression levels of costimulatory molecules were analyzed by flow cytometry.

### Analysis of GC and Tfh cells in NALT and cmLN

Groups of C57BL/6JNarl mice were intranasally administered with 20 µg of rDS, 20 µg of rDS plus 10 µg of rLF, 20 µg of rDS-F, or 20 µg of rDS-F plus 10 µg of rLF. At 12 days after administration, a single NALT cell suspension or cmLN cell suspension (pooled from three mice) was dissected out as mentioned in the preceding sections. Single cells were then incubated with anti-mouse CD16/CD32 antibody (Biolegend, Cat#101335, clone 93) for 30 min on ice to block nonspecific binding on Fc receptors. The Zombie Violet Fixable viability kit (Biolegend, Cat# 423113) was used to evaluate the viability of NALT and cmLN cells by flow cytometry. B cells and T cells were distinguished by staining with Alexa Fluor 488-conjugated anti-mouse/human B220 antibody (Biolegend, Cat# 103225, clone RA3-6B2) and Brilliant Violet 510-conjugated anti-mouse CD3 antibody (Biolegend, Cat# 100234, clone 17A2). GC B cells were distinguished by staining with Alexa Fluor 700-conjugated anti-CD38 antibody (Biolegend, Cat# 102742, clone 90) and PerCP/Cyanine5.5-conjugated anti-mouse/human GL-7 antibody (Biolegend, Cat# 144610, clone GL7). Additionally, biotin-conjugated rDS was added and then bound with PE-conjugated streptavidin to distinguish cells with antigen specificity. Tfh cells were distinguished by staining with PE/Cyanine7-conjugated anti-mouse CD4 antibody (Biolegend, Cat# 116016, clone RM4-4), Brilliant Violet 711-conjugated anti-mouse CD44 antibody (Biolegend, Cat# 103057, clone IM7), APC-conjugated anti-mouse PD-1 antibody (Biolegend, Cat# 109112, clone RMP1-30), and APC/Cyanine7-conjugated anti-mouse CXCR5 antibody (Biolegend, Cat# 145526, clone L138D7). The total cell numbers of the antigen-positive (Alexa Fluor 700^+^) GC B cells and Tfh cells were analyzed by flow cytometry.

### Mice immunization and sample collection

All intranasal administrations and blood sample collections were conducted under light sedation using isoflurane. Groups of mice (6–8 weeks of age) were vaccinated three times with 20 µg of rDS, 20 µg of rDS plus 10 µg of rF, 20 µg of rDS-F, or 20 µg of rDS-F plus 10 µg of rLF at a 2-week interval by intranasal administration. PBS was used as a control. Blood samples for serum collection were obtained using the submandibular vein method at time points as indicated. At 6 weeks after the first vaccination, mice were euthanized by controlled administration of carbon dioxide inhalation. Subsequently, BALF and NW samples were collected.

### Measurement of antibody titers

The antigen-specific IgG and IgA titers in the indicated samples were determined by titration as previously described (22) (Supplementary methods).

### Enzyme-linked immunospotassays

ELISPOT kits (BD Biosciences, San Jose, CA, USA, Cat#551083) were used according to the manufacturer’s instructions. The mice were euthanized using controlled administration of carbon dioxide inhalation 6 or 24 weeks after the first immunization and splenocytes were extracted. Splenocytes were isolated from the spleen using manual disruption, followed by the elimination of red blood cells using RBC Lysis Buffer. The splenocytes were predominantly cultured in complete RPMI medium (consisting of RPMI medium supplemented with 10%, vol/vol FBS, 25 mM HEPES, 1 mM sodium pyruvate, and penicillin-streptomycin). The splenocytes (5 × 10^5^ cells/well) were seeded into 96-well plates and incubated with 10 µg/mL CD4-specific or CD8-specific peptides derived from SARS-CoV-2 spike protein (38) for 3 days. Splenocytes incubated with 10 µg/mL control peptides or media (no stimulation) were severed as negative controls. The peptides, including S_62-76_ (VTWFHAIHVSGTNGT) peptides, S_263-270_ (AAYYVGYL) peptides, S_538-546_ (CVNFNFNGL), S_820-828_(DLLFNKVTL), CD4 control peptides (GRLITVNPIVTEKDS, derived from dengue virus), and CD8 control peptides (QYEGDGSPCKIPFEI, derived from dengue virus) were used. The spots were determined as previously described (21) (Supplementary Methods).

### Quantification of cytokine release

The spleens were removed to create single-cell suspensions at 5 weeks after the first immunization. Splenocytes were added to each well of a 24-well plate and further stimulated with rDS or BSA at a concentration of 10 µg/mL. Each stimulation was conducted in a separate well. After 3 days of culturing, cell-free supernatants were harvested and stored at −80°C. The IFN-γ, IL-5, IL-13, and IL-17A levels were measured using IFN gamma Mouse Uncoated ELISA Kit (Invitrogen, cat# 88–7314-88), IL-5 Mouse Uncoated ELISA Kit (Invitrogen, cat# 88–7054-88), IL-13 Mouse Uncoated ELISA Kit (Invitrogen, cat# 88-7137-88), and IL-17A (homodimer) Mouse Uncoated ELISA Kit (Invitrogen, cat# 88-7371-88) according to the manufacturer’s instructions.

### Intracellular staining and analysis of antigen-specific T_RM_ cells in the lung

The mice were immunized as described in the preceding sections, and their lung tissues were collected 5 weeks after immunization to create a single-cell suspension, as mentioned in the preceding sections. For intracellular cytokine analysis, the cells were incubated with rDS at 37°C and 5% CO_2_ for 18 hours and co-incubated with GolgiStop protein Transport Inhibitor (contains monensin and Brefeldin A) (BD Biosciences) during the last 6 hours. After two washes with PBS, the cells were incubated with anti-mouse CD16/CD32 antibody (Biolegend, Cat#101335, clone 93) for 30 min on ice to block nonspecific binding on Fc receptors. The cells were then stained with the Zombie Violet Fixable viability kit (Biolegend, Cat# 423113) to evaluate cell viability and labeled with surface markers for 30 min at 4°C in the dark, including Brilliant Violet 711-conjugated anti-CD3 antibody (Biolegend, Cat#100241, clone 17A2), PE/Cyanine7-conjugated anti-mouse CD4 antibody (Biolegend, Cat# 116016, clone RM4-4), PerCP/Cyanine5.5-conjugated anti-mouse CD8a antibody (Biolegend, Cat# 100734, clone 53-6.7), Brilliant Violet 510-conjugated anti-mouse CD62L antibody (Biolegend, Cat# 104441, clone MEL-14), Alexa Fluor 488-conjugated anti- mouse CD44 antibody (Biolegend, Cat# 103016, clone IM7), PE-conjugated anti-mouse CD69 antibody (Biolegend, Cat# 104508, clone H1.2F3), APC-conjugated anti-mouse CD11a antibody (Biolegend, Cat# 153110, clone I21/7), and Alexa Fluor 700-conjugated anti-mouse CD103 antibody (Biolegend, Cat# 121442, clone 2E7), to distinguish the CD4^+^ T_RM_ and CD8^+^ T_RM_ cells. Then, the cells were washed twice with staining buffer (PBS buffer containing 0.1% BSA and 0.05% sodium azide) and subjected to intracellular staining with IFN-γ, following the staining protocols provided by the manufacturer’s instructions for the BD Cytofix/Cytoperm TM kit (BD Biosciences). The cells were fixed and permeabilized with the Fixation/Permeabilization solution (BD Biosciences) for 20 min at 4°C, then washed twice with BD Per/WashTM buffer (BD Biosciences), and stained with stinging with APC/Cyanine7-conjugated anti-mouse IFN-γ antibody (Biolegend, Cat# 505850, clone XMG1.2) for 30 min. After two washes, the cells were resuspended in staining buffer prior to flow cytometric analysis.

### SARS-CoV-2 neutralization assay

The virus neutralization assay was conducted in a biosafety level 3 (BSL-3) laboratory and was approved by the Taiwan Centers for Disease Control (CDC). The strains of SARS-CoV-2, hCoV-19/Taiwan/4/2020 (Wuhan), hCoV-19/Taiwan/729/2020 (Alpha, B.1.1.7), hCoV/Taiwan/1013 (Beta, B.1.351), hCoV/Taiwan/1144/2020 (Delta, B.1.617.2), and hCoV-19/Taiwan/16804/2021 (Omicron, B.1.1.529) were obtained from the CDC in Taiwan. Viruses were amplified by the BSL3 team of National Health Research Institutes, Taiwan. The virus titer was determined by calculating the 50% tissue culture infectious dose (TCID_50_) using a standard method. Briefly, Vero cells were seeded (2.4 × 10^4^ cells/per well) in 96-well plates and cultured in M199 medium supplemented with 5% FBS at 37°C for 24 hours to form a monolayer. The next day, serum samples were incubated with 200 TCID_50_ of SARS-CoV-2 for 2 hours at 37°C. The serum samples were serially diluted in M199 medium in twofold dilutions starting from 1:20. The antibody-virus complexes were added to Vero cell culture monolayers in 96-well plates. The plates were incubated in a CO_2_ incubator at 37°C for 4 days, after which the cytopathic effect was observed microscopically. The neutralization titer was determined as the highest dilution of SARS-CoV-2 mAbs that prevented infection of 50% of quadruplicate inoculations.

### Syrian hamster immunization and challenge

Groups of hamsters (6–12 weeks of age) were vaccinated three times with 20 µg of rDS, 20 µg of rDS plus 10  µg of rLF, 20 µg of rDS-F, or 20 µg of rDS-F plus 10  µg of rLF at a 2-week interval by intranasal administration. PBS was used as a control. At 6 weeks after first immunization, Syrian hamsters were challenged intranasally with 1 × 10^4^ TCID_50_ (Wuhan, Delta, and Omicron) SARS-CoV-2 in 50 µL under isoflurane anesthesia. Half of the hamsters in each group were sacrificed on day 3 after the challenge to quantify the viral load. Left lung tissues were homogenized in 2 mL of PBS using a gentleMACS Dissociator (Miltenyi Biotec) to determine the viral load in the lung. The other half of the hamsters in each group were weighed daily until day 6 after the challenge and euthanized by controlled administration of carbon dioxide inhalation for pathological analysis of lung lobes. Lung tissues collected at necropsy were fixed in 10% PBS buffered formaldehyde for 24 hours, then processed into paraffin-embedded tissue blocks. The 5-µm sections were stained with hematoxylin and eosin for histopathological examinations. Lung tissues were fixed with 4% paraformaldehyde and processed for paraffin embedding. Images were captured using a Leica DFC 5400 digital camera and were processed using Leica Application Suite v.4.13. The mock tissue sections in Fig. 9D and E are identical and for reference only. The first image of the mock tissue section is adapted from our previous study (40). Pathological severity scores were evaluated according to the percentage of inflammation area for each section from each animal using the following scoring system: 0, no pathological change; 1, affected area ≤ 10%; 2, affected area 10%–30%; 3, affected area 30%–50%; and 4, affected area ≥ 50%.

### Statistical analysis

If applicable, the ordinary one-way ANOVA followed by Tukey’s multiple comparison test was given priority to compare differences among more than two groups. Otherwise, the Kruskal-Wallis test with Dunn’s multiple comparison test was used. Statistical analysis was performed using GraphPad Prism software version 6.01 (GraphPad Software, San Diego, CA, USA). Differences with *P* < 0.05 were considered statistically significant.

## SUPPLEMENTAL MATERIAL

Supplemental material jvi.01546-23-s0001.pdf Figures S1 to S3, Table S1, and supplemental methods.

ASM does not own the copyrights to Supplemental Material that may be linked to, or accessed through, an article. The authors have granted ASM a non-exclusive, world-wide license to publish the Supplemental Material files. Please contact the corresponding author directly for reuse.
