# Supplementary material for: SARS-CoV-2 spike-FLIPr fusion protein plus lipidated FLIPr protects against various SARS-CoV-2 variants in hamsters
Source: J Virol. 2024 Feb 1;98(2):e01546-23. doi: 10.1128/jvi.01546-23 (PMC10878263; doi:10.1128/jvi.01546-23)
Supplement: Supplemental material — Figures S1 to S3, Table S1, and supplemental methods. [file jvi.01546-23-s0001.pdf]

Supplementary Materials for

SARS-CoV-2 spike-FLIPr fusion protein plus lipidated FLIPr protects against various SARS-CoV-2 variants in hamsters

Ming-Shu Hsieh,[1] Chia-Wei Hsu,[1] Hung-Chun Liao,[1] Chang-Ling Lin,[1] Chen-Yi Chiang,[1] Mei-Yu Chen,[1] Shih-Jen Liu,[1,2,3] Ching-Len Liao[1] and Hsin-Wei Chen[1,2,3*]

[1] National Institute of Infectious Diseases and Vaccinology, National Health Research Institutes, Miaoli, Taiwan.

[2] Graduate Institute of Biomedical Sciences, China Medical University, Taichung, Taiwan.

[3] Graduate Institute of Medicine, Kaohsiung Medical University, Kaohsiung, Taiwan.

*Correspondence:
Hsin-Wei Chen (Email: chenhw@nhri.org.tw)

Included:
Supplementary Figure 1-3
Supplementary Table 1
Supplementary Methods

Figure S1

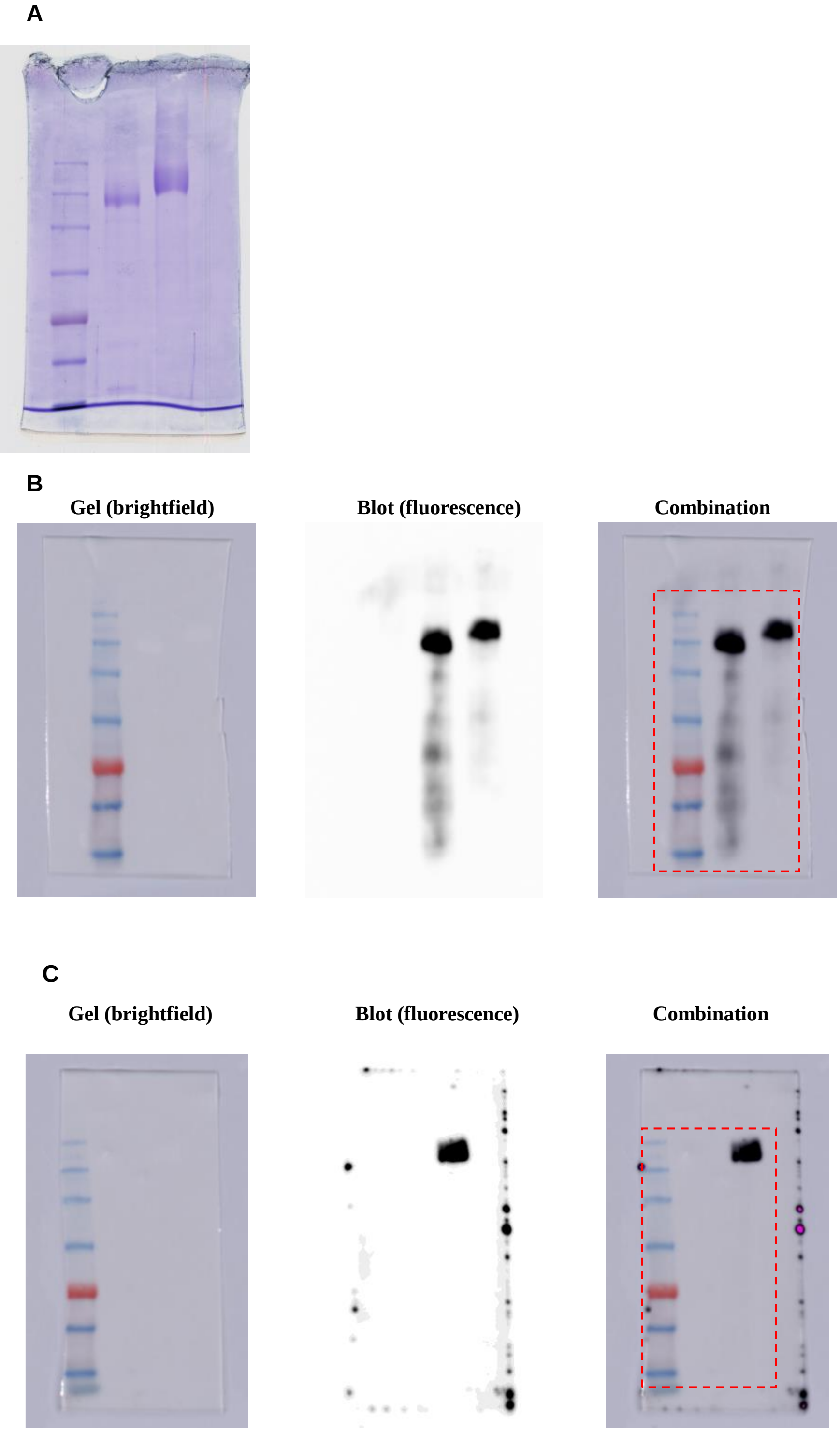

Figure S1. Raw images for Supplementary Figure S1. (A) the left panel (B) the middle panel (C) the right panel. Red dotted lines specify each area which was cropped to generate respective panels in supplementary Figure S1A as indicated.

Figure S2

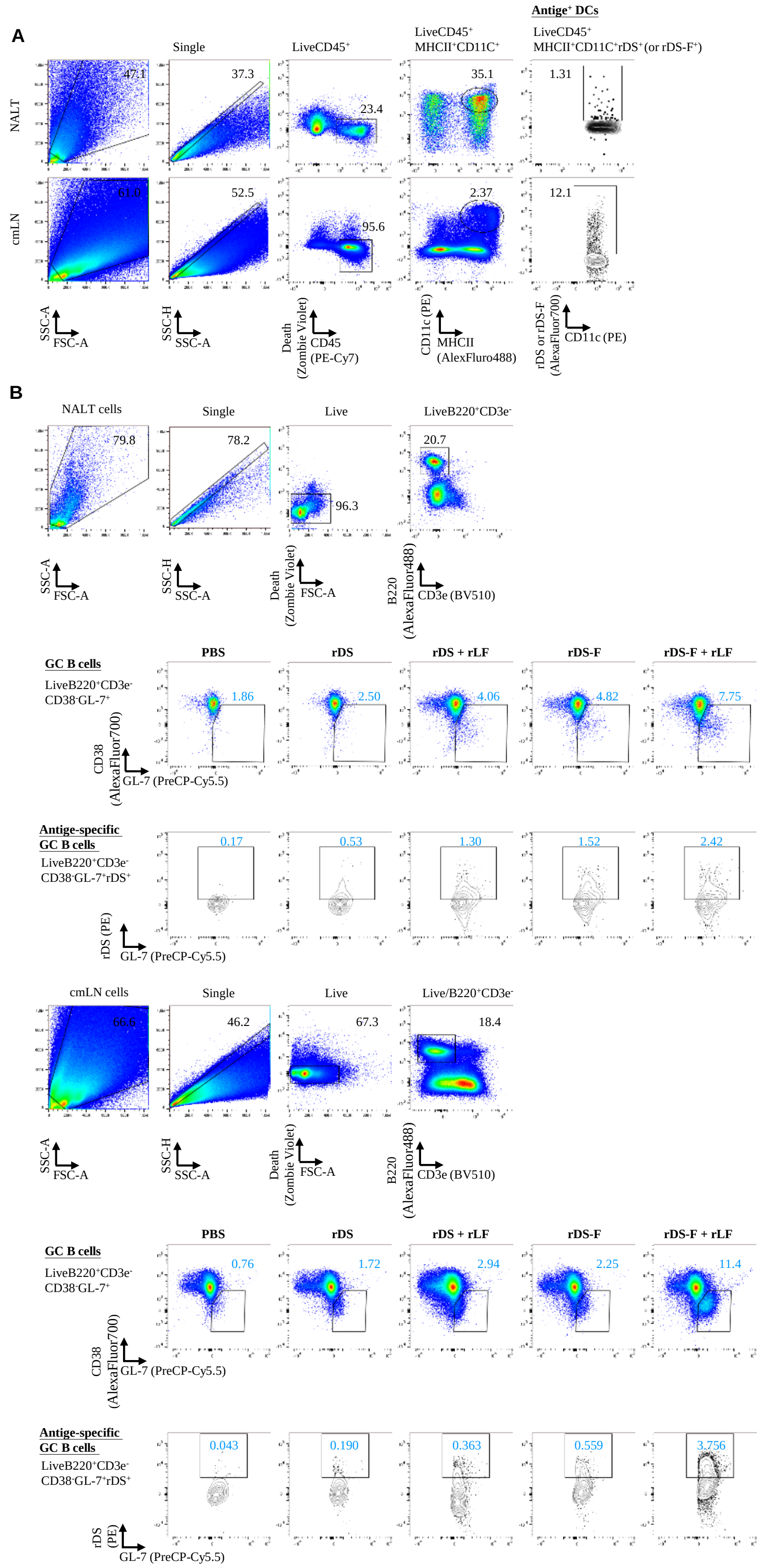
A
Antige⁺ DCs
Single
LiveCD45⁺
LiveCD45⁺ MHCII⁺CD11C⁺
LiveCD45⁺ MHCII⁺CD11C⁺rDS⁺(or rDS-F⁺)
NALT
cmLN
47.1
37.3
23.4
35.1
1.31
61.0
52.5
95.6
2.37
12.1
SSC-A
FSC-A
SSC-H
SSC-A
Death (Zombie Violet)
CD45 (PE-Cy7)
CD11c (PE)
MHCII (AlexFluro488)
rDS or rDS-F (AlexaFluor700)
CD11c (PE)
B
NALT cells
Single
Live
LiveB220⁺CD3e⁻
79.8
78.2
96.3
20.7
SSC-A
FSC-A
SSC-H
SSC-A
Death (Zombie Violet)
FSC-A
B220 (AlexaFluor488)
CD3e (BV510)
PBS
rDS
rDS + rLF
rDS-F
rDS-F + rLF
GC B cells
LiveB220⁺CD3e⁻ CD38⁻GL-7⁺
1.86
2.50
4.06
4.82
7.75
CD38 (AlexaFluor700)
GL-7 (PreCP-Cy5.5)
Antige-specific GC B cells
LiveB220⁺CD3e⁻ CD38⁻GL-7⁺rDS⁺
0.17
0.53
1.30
1.52
2.42
rDS (PE)
GL-7 (PreCP-Cy5.5)
cmLN cells
Single
Live
Live/B220⁺CD3e⁻
66.6
46.2
67.3
18.4
SSC-A
FSC-A
SSC-H
SSC-A
Death (Zombie Violet)
FSC-A
B220 (AlexaFluor488)
CD3e (BV510)
PBS
rDS
rDS + rLF
rDS-F
rDS-F + rLF
GC B cells
LiveB220⁺CD3e⁻ CD38⁻GL-7⁺
0.76
1.72
2.94
2.25
11.4
CD38 (AlexaFluor700)
GL-7 (PreCP-Cy5.5)
Antige-specific GC B cells
LiveB220⁺CD3e⁻ CD38⁻GL-7⁺rDS⁺
0.043
0.190
0.363
0.559
3.756
rDS (PE)
GL-7 (PreCP-Cy5.5)

C

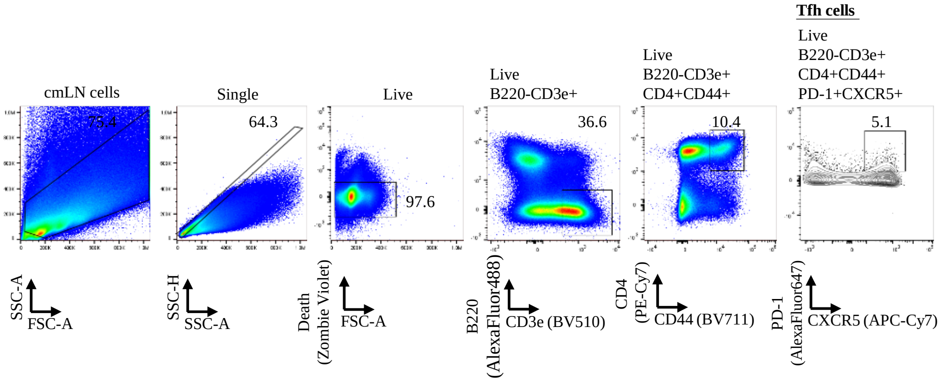

Figure S2. Gating strategies for DCs, GC B cells and Tfh cells are indicated. The gating strategy for identification of (A) DCs (gated on liveCD45$^+$CD11c$^+$MHCII$^+$), (B) antigen-specific GC B cells (gated on liveB220$^+$CD3e$^-$CD38$^-$GL-7$^+$rDS$^+$) in NALT and cmLN, and (C) Tfh cells (gated on liveB220$^-$CD3e$^+$CD4$^+$CD44$^+$PD-1$^+$CXCR5$^+$) in cmLN are shown. The black font numbers adjacent to the contour regions indicate the percentage of the current total cell count in the gating compared to the total cell count in the preceding gating. The numbers in light blue font in figure (B) indicate the percentage of cells in the current gating out of the total B cells. An example of a representative samples is shown.

Figure S3

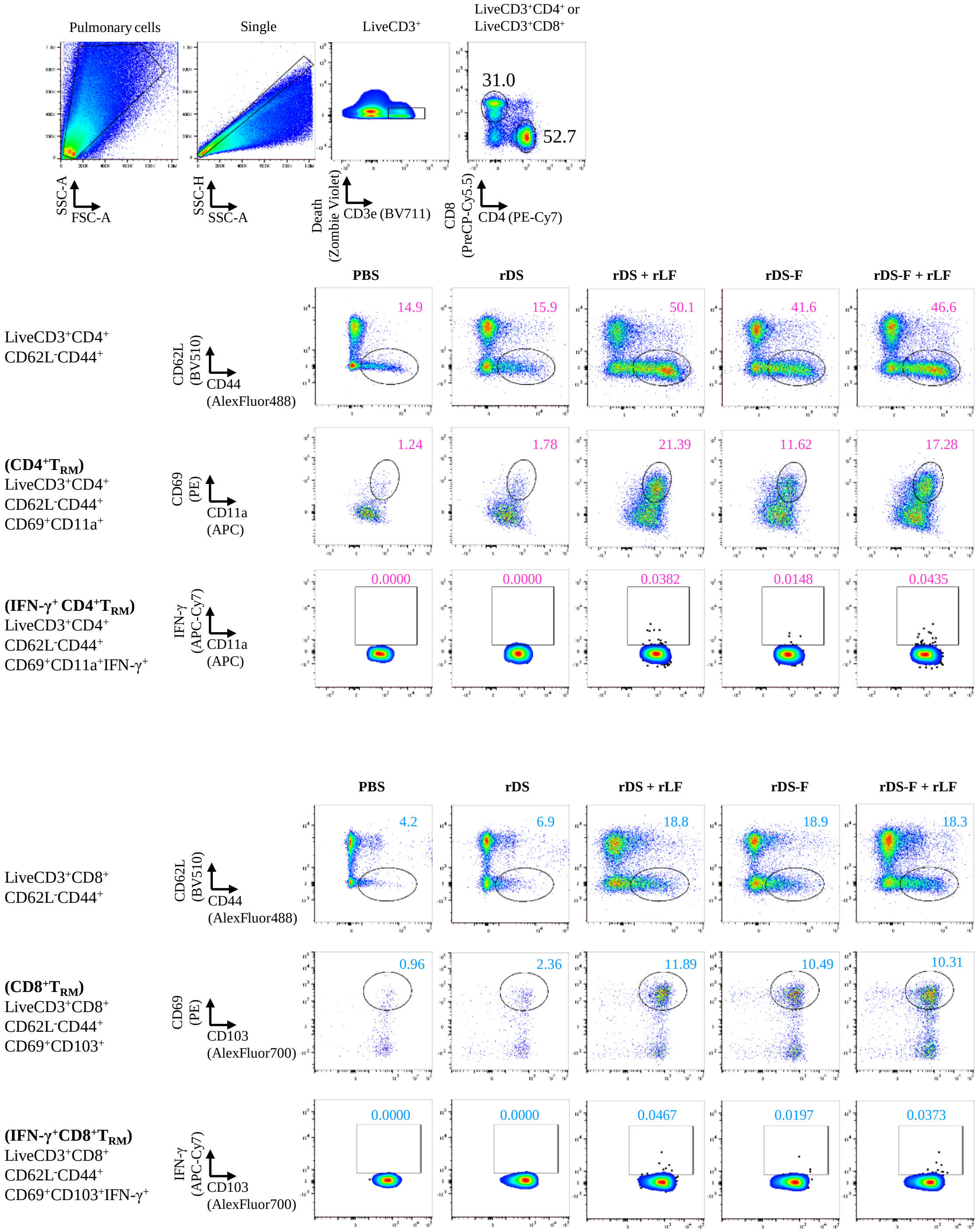
Pulmonary cells
Single
LiveCD3+
LiveCD3+CD4+ or
LiveCD3+CD8+
31.0
52.7
SSC-A
FSC-A
SSC-H
SSC-A
Death
(Zombie Violet)
CD3e (BV711)
CD8
(PreCP-Cy5.5)
CD4 (PE-Cy7)
PBS
rDS
rDS + rLF
rDS-F
rDS-F + rLF
LiveCD3+CD4+
CD62L-CD44+
CD62L
(BV510)
CD44
(AlexFluor488)
14.9
15.9
50.1
41.6
46.6
(CD4+TRM)
LiveCD3+CD4+
CD62L-CD44+
CD69+CD11a+
CD69
(PE)
CD11a
(APC)
1.24
1.78
21.39
11.62
17.28
(IFN-γ+ CD4+TRM)
LiveCD3+CD4+
CD62L-CD44+
CD69+CD11a+IFN-γ+
IFN-γ
(APC-Cy7)
CD11a
(APC)
0.0000
0.0000
0.0382
0.0148
0.0435
PBS
rDS
rDS + rLF
rDS-F
rDS-F + rLF
LiveCD3+CD8+
CD62L-CD44+
CD62L
(BV510)
CD44
(AlexFluor488)
4.2
6.9
18.8
18.9
18.3
(CD8+TRM)
LiveCD3+CD8+
CD62L-CD44+
CD69+CD103+
CD69
(PE)
CD103
(AlexFluor700)
0.96
2.36
11.89
10.49
10.31
(IFN-γ+CD8+TRM)
LiveCD3+CD8+
CD62L-CD44+
CD69+CD103+IFN-γ+
IFN-γ
(APC-Cy7)
CD103
(AlexFluor700)
0.0000
0.0000
0.0467
0.0197
0.0373

Figure S3. Gating strategies for IFN-γ^+ CD4$^+$ T_{RM} cells and IFN-γ^+ CD8$^+$ T_{RM} cells are indicated. The gating strategy for identifying IFN-γ^+ CD4$^+$ T_{RM} cells (gated on liveCD3$^+$CD4$^+$CD62L$^-$CD44$^+$CD69$^+$CD11a$^+$IFN-γ^+) and IFN-γ^+ CD8$^+$ T_{RM} cells (gated on liveCD3$^+$CD8$^+$CD62L$^-$CD44$^+$CD69$^+$ CD103$^+$IFN-γ^+) in lung tissue is shown as an example of representative samples. The percentages of total cells in the current gating compared to the previous gating are indicated by the numbers in black font adjacent to the outlined areas. The percentages of cells in the current gating out of the total CD4$^+$ T cells or CD8$^+$ T cells are marked in pink or light blue font, respectively.

Supplementary Table 1.

The CD4-specific and CD8-specific epitopes used in this study were aligned among SARS-CoV-2 variants.

	S_{62-76}	$S_{263-270}$	$S_{538-546}$	$S_{820-828}$
Delta B.1.617.2	VTWFHAIHVSGTNGT	AAYYVGYL	CVNFNFNGL	DLLFNKVTL
Wuhan	VTWFHAIHVSGTNGT	conserved	conserved	conserved
Alpha B.1.1.7	VTWFHAI--SGTNGT	conserved	conserved	conserved
Beat B.1.35	VTWFHAIHVSGTNGT	conserved	conserved	conserved
Omicron B.1.1.529	VTWFHVI--SGTNGT	conserved	conserved	conserved

Note: The amino acid changes relevant to the SARS-CoV-2 Delta B.1.617.2 reference strain are shown in red font, while dashes (–) indicate gaps in the alignment. When we use the term 'conserved,' it means that the sequence is identical to the consensus sequence of SARS-CoV-2 variants.

Supplementary Methods

Production and purification of rLF

To express the rLF, the *Escherichia coli* C43(DE3) strain was transformed with pLF. The transformed cells were cultured with LB broth at 37 °C overnight. The overnight culture was scaled up by 50 times the original volume in a 2 L shake flask and incubated at 37 °C until the OD_{600} reached 0.6. Protein expression was induced (OD_{600} = 0.6) by adding 1 mM IPTG, followed by incubation at 20 °C for 24 h. rLF was purified by disrupting the harvested cells in a French press (Constant Systems, Daventry, UK) at 27 Kpsi in homogenization buffer [20 mM Tris (pH 8.0), 40 mM sucrose, 400 mM NaCl and 10% glycerol]. The cell lysate was clarified by centrifugation at 32,000 rpm for 40 min at 4 °C. Most of the rLF was present in inclusion bodies. rLF was then solubilized with extraction buffer [10 mM Na_2HPO_4 (pH 9.0) and 1% Triton X-100]. The extracted fraction was loaded onto immobilized metal affinity chromatography (IMAC) columns (2.5 cm i.d. × 10.0 cm) (BIO-RAD, Hercules, CA) containing 20 ml of Ni-NTA resin (Qiagen) to purify rLF. The column was washed with the extraction buffer and the same buffer containing 20 mM imidazole. Then, the rLF was eluted with homogenization buffer containing 500 mM imidazole. The eluted rLF was dialyzed to 20 mM Tris (pH 8.0) three times for at least 6 h each time. After dialysis, the rLF was loaded onto a 20-ml Q Sepharose fast flow column (GE Healthcare, Little Chalfont, Buckinghamshire, UK). The column was washed with dialysis buffer containing 200 mM NaCl and then washed with a 100-fold column volume of dialysis buffer containing 0.1% Triton X-114 to remove the lipopolysaccharide. Next, the column was washed without 0.1% Triton X-114 to remove the residual detergent, and rLF was eluted with elution buffer [10 mM Na_2HPO_4 (pH 9.0), 300 mM NaCl and 8 M urea]. The eluted rLF was dialyzed to 10 mM Na_2HPO_4 (pH 9.0) three times for at least 6 h each time.

The endotoxin levels of the purified rLF were determined using the limulus amebocyte lysate (LAL) assay (Associates of Cape Cod, Inc., Cape Cod, MA), and the resulting endotoxin levels were <30 EU/mg. After dialysis, the rLF were lyophilized and stored at −20 °C. The fractions from each step were analyzed by SDS-PAGE and immunoblotted with anti-FLIPr and anti-His tag antibodies. The N-terminal fragments of rLF were obtained and identified after trypsin digestion of rLF. The identification of the lipid moiety in rLF was performed on a Waters® MALDI micro MX™ mass spectrometer and described previously [21].

NALT single-cell preparation.

The NALT tissue was mechanically disrupted and transferred to conical tubes. Subsequently, tissue pieces were resuspended in 1 mL of RPMI supplemented with 0.4 mg/mL collagenase (Sigma-Aldrich, St. Louis, MO) and incubated at 37°C for 30 minutes. The tissue was then ground, and the resulting suspension was collected and filtered through a 70 µm cell strainer. The cells were collected by centrifugation at 300×g for 5 minutes.

Measurement of antibody titers.

Serum samples were serially diluted three-fold (starting at the indicated dilution) and added to 96-well plates coated with the antigen. 1:5000 diluted peroxidase-conjugated anti-mouse IgG Fc IgG fraction antibody (MP Biomedicals, cat#0855554) and 1:1500 diluted HRP Goat anti-Mouse IgA Cross-Adsorbed Secondary Antibody (Invitrogen, cat# 62-6720) were used to detect bound IgG and IgA, respectively. After washing with PBS, a substrate 3,3',5,5'-tetramethylbenzidine was added, and the absorbance at 450 nm was measured using an ELISA reader. The endpoint titer was determined as twice the mean of the background OD value. Titers were calculated by interpolation from the titration curve, unless the OD value was less than twice the mean of the

background at the starting dilution.

Spots determined of ELISPOT assays.

After incubation, the splenocytyes were discarded from the plates by washing three times with 0.05% (w/v) Tween 20 in PBS. The wells were first treated with biotinylated detection antibody (0.1 ml/well) and incubated at 37°C for 2 hours. The plates were then washed following the same steps as before and the avidin-horseradish peroxidase complex reagent was added. After incubating the plates at room temperature for 45 minutes, the wells were washed three times with 0.05% (w/v) Tween 20 in PBS followed by three washes with PBS alone. To develop the spots, staining solution (3-amine-9-ethylcarbazole, Sigma-Aldrich) was added to the wells (0.1 ml/well), and the plates were incubated for 1 hour. The reaction was stopped by placing the plates under tap water, and the spots were determined using an ELISPOT reader (Cellular Technology Ltd., Shaker Heights, OH, USA).
